# Gene expression in tonsils in swine following infection with porcine reproductive and respiratory syndrome virus

**DOI:** 10.1186/s12917-021-02785-1

**Published:** 2021-02-22

**Authors:** Qian Dong, Joan K. Lunney, Kyu-Sang Lim, Yet Nguyen, Andrew S. Hess, Hamid Beiki, Raymond R. R. Rowland, Kristen Walker, James M. Reecy, Christopher K. Tuggle, Jack C. M. Dekkers

**Affiliations:** 1 Department of Animal Science, Iowa State University, Ames, Iowa 50011 USA; 2 USDA, ARS, BARC, APDL, Beltsville, MD 20705 USA; 3 Department of Statistics, Iowa State University, Ames, Iowa 50011 USA; 4 College of Veterinary Medicine, University of Illinois at Urbana-Champaign, Urbana, IL 61802 USA

**Keywords:** PRRSV, Persistence, Tonsils, Pig, Isolate, WUR, RNA-seq, Cell composition

## Abstract

**Background:**

Porcine reproductive and respiratory syndrome (PRRS) is a threat to pig production worldwide. Our objective was to understand mechanisms of persistence of PRRS virus (PRRSV) in tonsil. Transcriptome data from tonsil samples collected at 42 days post infection (dpi) were generated by RNA-seq and NanoString on 51 pigs that were selected to contrast the two PRRSV isolates used, NVSL and KS06, high and low tonsil viral level at 42 dpi, and the favorable and unfavorable genotypes at a genetic marker (WUR) for the putative PRRSV resistance gene *GBP5*.

**Results:**

The number of differentially expressed genes (DEGs) differed markedly between models with and without accounting for cell-type enrichments (CE) in the samples that were predicted from the RNA-seq data. This indicates that differences in cell composition in tissues that consist of multiple cell types, such as tonsil, can have a large impact on observed differences in gene expression. Based on both the NanoString and the RNA-seq data, KS06-infected pigs showed greater activation, or less inhibition, of immune response in tonsils at 42 dpi than NVSL-infected pigs, with and without accounting for CE. This suggests that the NVSL virus may be better than the KS06 virus at evading host immune response and persists in tonsils by weakening, or preventing, host immune responses. Pigs with high viral levels showed larger CE of immune cells than low viral level pigs, potentially to trigger stronger immune responses. Presence of high tonsil virus was associated with a stronger immune response, especially innate immune response through interferon signaling, but these differences were not significant when accounting for CE. Genotype at WUR was associated with different effects on immune response in tonsils of pigs during the persistence stage, depending on viral isolate and tonsil viral level.

**Conclusions:**

Results of this study provide insights into the effects of PRRSV isolate, tonsil viral level, and WUR genotype on host immune response and into potential mechanisms of PRRSV persistence in tonsils that could be targeted to improve strategies to reduce viral rebreaks. Finally, to understand transcriptome responses in tissues that consist of multiple cell types, it is important to consider differences in cell composition.

**Supplementary Information:**

The online version contains supplementary material available at 10.1186/s12917-021-02785-1.

## Background

Porcine respiratory and reproductive syndrome (PRRS) is the most economically significant disease in modern pig production worldwide and has been estimated to cost $664 million annually in combined production losses in breeding and growing-pig herds in the United States [1] and about $793,000 per average European farm that was severely affected in breeding, nursery, and fattening stages [2]. The PRRS disease is caused by the PRRS virus (PRRSV), which is a positive single-strand, enveloped RNA virus that belongs to the order *Nidovirales*, family *Arteriviridae* [3]. PRRSV can modulate host immune response by inhibiting natural killer cell cytotoxic activity [4] and IFNα production [5] and by stimulating the negative regulator interleukin-4 [6], which can result in a weak and slow immune response and poor control of virus replication. This weak antiviral immune response can lead to a persistent infection in a subset of pigs, which can cause a second outbreak when the virus is transmitted to naïve pigs through oral-nasal secretions and semen [7]. In asymptomatic carrier pigs, PRRSV can persist in lymphoid tissues for 251 days, including tonsils and lymph nodes [8, 9]. In addition, clinical signs of PRRS vary greatly between pigs and between PRRSV strains, which is primarily due to the genetic diversity within the structural and non-structural proteins of the virus that may result from mutations during viral replication in the host, all of which contribute to the difficulty to control PRRSV with vaccines [10]. Thus, more knowledge is needed about the mechanisms of host resistance and viral persistence during PRRSV infection in pigs.

The PRRS Host Genetics Consortium (PHGC) was established in 2007, with the aim to investigate the genetic basis of host response to PRRSV infection in nursery pigs [11]. Results showed that host response to PRRSV is moderately heritable but highly polygenic [12], beyond the effects of a putatively causative intronic SNP, rs340943904, in the guanylate binding protein 5 gene (*GBP5*), which results in an early stop codon and a putatively non-functional GBP5 protein [13]. This SNP was found to be in complete linkage disequilibrium with SNP rs80800372 (WUR) on *Sus scrofa* chromosome 4 [13]. The effect of the WUR SNP on viral load (VL) and weight gain (WG) after PRRSV infection was confirmed for two genetically distinct North American PRRSV isolates, NVSL-97-7895 (NVSL) and KS-2006-72109 (KS06), for vaccination with a PRRS modified live vaccine (MLV), for co-infection with PRRSV and PCV2b [14–16], and for infection with an attenuated European PRRSV strain (effect on growth rate only) [17]. Results from the same PHGC trials as used in the present study have shown that the NVSL strain is more virulent and less persistent in serum than the KS06 strain, and that pigs with the AB or BB genotype at the WUR SNP have significantly lower serum viremia than AA pigs following infection with either PRRSV isolate [16]. The effect of WUR genotype on PRRSV persistence, however, differed depending on virulence of the PRRSV isolate [16]. Moreover, anti-viral immune responses of pigs differed substantially between PRRSV isolates [18, 19]. Therefore, it is important to understand the mechanisms of host response following infection with different PRRSV isolates to help develop intervention strategies, to improve anti-viral responses, and to clear PRRSV infection.

Previous studies have investigated the genetic basis of host response during the acute stage of PRRSV infection, but little is known about the genetic basis of PRRSV persistence. Guo et al. [20] found that a higher level of double-stranded RNA (dsRNA) of the PRRSV in the germinal center of lymph nodes, a mediator for viral persistence, did not stimulate antiviral immunity. In addition, using data from five PHGC trials, including the two trials used here, Hess et al. [21] reported that low tonsil viral level at 42 days post infection (dpi) was phenotypically associated with an earlier and faster rate of maximal viral clearance from blood, with lower viral load in serum from 0 to 42 dpi, and with lower serum viremia at 42 dpi. Abella et al. [17] suggested that pigs that were non-viremic between 4 and 42 days post vaccination did not become a reservoir for an attenuated European PRRSV strain in tonsils. Furthermore, Lunney et al. [22] showed that variation in the levels of three serum cytokines (IL-8, IL-1β, and IFN-γ was significantly correlated with serum virus levels from 1 to 21 dpi, accounting for 84% of the variation in virus levels in lymphoid tissues between pigs, including inguinal lymph nodes, submaxillary lymph nodes, and tonsils. To better understand mechanisms of PRRSV persistence in lymphoid tissues, especially in tonsils, we need to know more about the genes associated with host response to PRRSV in tonsils.

Different immune cell types in tonsils may have individual roles in developing innate, cellular, and/or humoral immunity. For example, tonsils contained a higher percentage of B cells than blood and some other lymphoid tissues [23], and the percentages of antiviral antibody secreting B cells in tonsils increased significantly from 3 to 60 days after PRRSV infection [24]. Kawashima et al. [25] also observed differences in the numbers of CD4+ T cells, CD8+ T cells, and B cells in tonsils from 0 to 10 dpi after PRRSV inoculation of 53 to 55 day-old pigs. Generally, the number of B cells in tonsils increases and the numbers of CD4+ T cells and CD8+ T cells decreases until 10 days after PRRSV infection [25]. This emphasizes the importance of considering cell composition of tonsils during PRRSV infection.

The objectives of this study were to investigate: (1) mechanisms of PRRSV persistence in nursery pigs by studying the tonsil transcriptome at 42 days after infection with one of two PRRSV isolates, NVSL and KS06; (2) the effect of PRRSV level on the tonsil transcriptome at 42 dpi; and (3) the effect of genotype at a genetic marker for the *GBP5* resistance gene (WUR) on the tonsil transcriptome at 42 dpi. Our hypotheses were that (1) the more virulent NVSL PRRSV isolate inhibits host immunity in the tonsil more, or activates it less, than the less virulent KS06 PRRSV isolate; (2) a higher virus level in the tonsil induces a stronger immune response; and (3) pigs with the favorable AB genotype at WUR (and *GBP5*) induce a stronger innate immune response than AA pigs. Data and samples from two PHGC trials were used, noting that these trials were designed to evaluate variation in host response to PRRSV infection between pigs. Thus, contemporary negative controls without infection were not available.

## Results

### Viral levels and sample selection

**Fig. 1:**
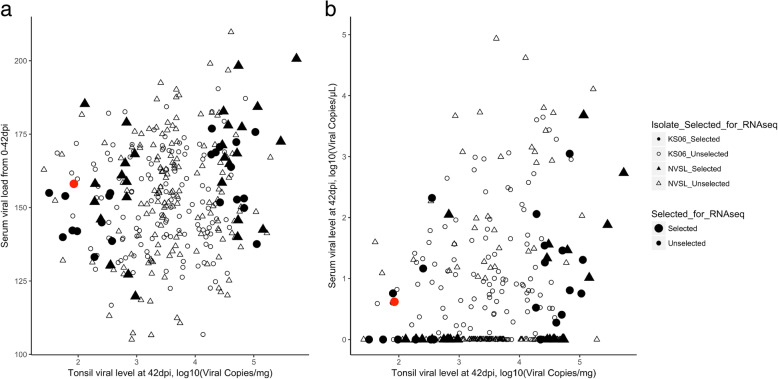
The relationship between tonsil and serum viral level in pigs infected with the NVSL or KS06 PRRS virus isolate. **a** Serum viral load, calculated as area under the Wood’s curve from the day of infection until euthanasia at 42 days post infection (dpi), compared to viral level in tonsil at 42 dpi. Solid black symbols indicate samples selected for RNA-sequencing and NanoString gene expression analyses (except one sample noted in red, which was used only for RNA-seq). The correlation of tonsil viral level at 42 dpi with VLTotal was 0.14 (*p* = 0.01). **b** Serum viremia level at 42 dpi compared to tonsil viral level at 42 dpi. Of the 337 pigs, 168 had a non-zero tonsil viral level (non-zero on the x axis), although they had non-detectable levels of serum viremia at 42 dpi (zero on the y axis), 80.9% of the NVSL-infected pigs and 22.8% of the KS06-infected pigs. A total of 53 tonsil samples (shown as “Selected”) were selected for gene expression analyses based on high or low PRRSV level (at least one standard deviation from the mean) in the tonsil (High and Low-TVclass) and RIN scores of the extracted RNA

Across all pigs in the two PHGC trials used here, the NVSL-infected pigs (trial PHGC5) numerically had higher viral load in serum from 0 to 42 dpi (VLTotal) than the KS06-infected pigs (trial PHGC14) (Fig. 1a) but this difference was not significant. The correlation of tonsil viral level at 42 dpi (adjusted for the use of different kits for the semi-quantitative TaqMan PCR assay for PRRSV RNA [21]) and VLTotal was 0.14 (*p* = 0.01, Fig. 1a). Although 168 of the 337 infected pigs (80.9% of the 180 NVSL-infected pigs and 22.8% of the 157 KS06-infected pigs) had no detectable viremia levels at 42 dpi, PRRSV RNA could still be detected in tonsils at 42 dpi for all pigs (Fig. 1b). The NVSL-infected pigs (trial 1, *n* = 180) had significantly (*p* = 0.02) higher tonsil viral level at 42 dpi than the KS06-infected pigs (trial 2, *n* = 157) (3.65 ± 0.06 versus 3.43 ± 0.07 log_10_(Viral Copies/mg), Figure S1). However, the NVSL-infected pigs had significantly (*p* = 0.01) lower serum virus levels at 42 dpi (V42dpi) than the KS06-infected pigs (1.40 ± 0.09 versus 0.95 ± 0.08 log_10_(Viral Copies/μL)). Figure S1 also identifies the 51 tonsil samples that were selected for gene expression analyses based on high versus low tonsil viral level (TVclass), as well as the RNA integrity number (RIN) score of the samples, as a measure of RNA integrity.

### Tonsil Transcriptome alignment and mapping

After excluding two samples with small library size (179,735 and 456,204 reads), more than 1.5 billion (1,573,259,257) 100-base paired-end reads were produced by RNA-seq across the 51 samples. The number of reads per sample averaged 30,848,221 and ranged from 967,422 to 71,136,944, comprising on average 3.5 gigabases of sequence data per sample. On average, 97.5% of reads were mapped to build 11.1 of the pig reference genome, of which 36.7% were uniquely mapped and 60.9% were multiple mapping reads. Based on the uniquely mapped reads, 24,432 genes had an average read count larger than zero across all samples, accounting for about 63.7% of all 38,371 annotated genes, including 25,880 Ensembl genes and 12,491 “novel genes” that were identified in the single-molecule long-read isoform sequencing (Iso-Seq) data by Beiki et al. [26]. Genes with an average read count less than eight and with non-zero mapped reads in fewer than four samples were removed. In total, 19,107 genes were determined to be expressed in tonsil, of which 12,243 were annotated in Ensembl (47.3% of the 25,880 genes in Ensembl) and 6863 were novel genes from Iso-Seq (54.9% of the 12,491 novel genes from Iso-Seq), including the *GBP5* gene.

### Factors affecting cell enrichments in tonsils

The tonsil RNA-seq data were analyzed using the xCell software [27] to quantify cell type enrichments (CE) and to produce an aggregated immune score for each sample (the sum of scores of B cells, CD4+ T cells, CD8+ T cells, DC, eosinophils, macrophages, monocytes, mast cells, neutrophils, and NK cells, divided by 1.5). Pigs with a high virus level in tonsil (High-TVclass) had significantly larger CE scores for immune cells per unit of library size of the tonsil RNA-seq data than pigs with a low virus level in tonsil (Low-TVclass) (*p* = 0.034) (Table 1). In addition, although not significant (*p* = 0.27 and 0.46, respectively), tonsils from KS06-infected pigs and from AB genotype pigs had numerically larger CE scores for immune cells than tonsils from NVSL-infected and AA genotype pigs, respectively. For lymphoid and myeloid cells and their subsets, tonsils from KS06-infected pigs, from High-TVclass pigs, and from AA genotype pigs had significantly (*p* < 0.1) larger CE scores of specific immune cells (except plasmacytoid dendritic cells and naïve B-cells) than tonsils from NVSL-infected pigs, from High-TVclass pigs, and from AB genotype pigs, respectively (Table 1).

**Table 1:** Lymphocytes and myeloid cells with significant effects of the factors of Isolate, TVclass, or WUR genotype based on xCell enrichment scores

Full name		dLSM^a^	SE	p-value	Cell group
**KS06-NVS**L					
Isolate	Class-switched memory B-cells	0.013	0.006	0.032	Lymphocytes
	CD4+ central memory T-cells	0.003	0.002	0.098	Lymphocytes
	Basophils	0.042	0.017	0.016	Myeloid cells
	Plasmacytoid dendritic cells	− 0.003	0.002	0.099	Myeloid cells
**High-Low**					
TVclass	B-cells	0.021	0.012	0.081	Lymphocytes
	CD8+ T-cells	0.011	0.006	0.059	Lymphocytes
	Class-switched memory B-cells^c^	0.017	0.005	0.004	Lymphocytes
	Memory B-cells	0.009	0.004	0.042	Lymphocytes
	naive B-cells	−0.004	0.002	0.048	Lymphocytes
	Plasma cells	0.008	0.003	0.028	Lymphocytes
	Type 1 T-helper cells	0.007	0.003	0.027	Lymphocytes
	CD8+ naive T-cells	0.005	0.002	0.033	Lymphocytes
	CD8+ central memory T-cells^c^	0.021	0.007	0.003	Lymphocytes
	Basophils^c^	0.046	0.016	0.006	Myeloid cells
	Activated dendritic cells	0.016	0.008	0.048	Myeloid cells
	Plasmacytoid dendritic cells	0.005	0.002	0.020	Myeloid cells
	Immune score^b^	0.021	0.010	0.034	
**AA-AB**					
WUR	Type 1 T-helper cells	0.007	0.004	0.082	Lymphocytes
	Plasmacytoid dendritic cells	0.005	0.002	0.056	Myeloid cells

^a^ Differences of least squared means (dLSM) between factor levels for cell enrichment scores: LSM for KS06 minus LSM for NVSL isolates; High minus Low tonsil viral class; AA minus AB WUR genotypes. Only estimates of significance (*p* < 0.1) for lymphocytes and myeloid cells and their subsets are shown based on the library size of the RNA-seq data

^b^ Immune score, as determined by the xCell software, is an aggregated score that considers B cells, CD4+ T cells, CD8+ T cells, dendritic cells, eosinophils, macrophages, monocytes, mast-cells, neutrophils, and natural killer cells

^c^Significant after multiple test correction

After analyzing the 64 cell types in xCell for the effects of Isolate, TVclass, WUR, age, sex, RIN, and two-way interactions among Isolate, TVclass, and WUR, the CE scores of 28 cell types were found to be significantly affected (*p* < 0.05) by at least one of these factors and were put in the significant (Sig) group of cell types (Table S1), while the remaining 34 cell types were allocated to the nuisance (Nui) group. In the Sig group, 14 of 28 cell types were immune cells, compared to 20 of 34 cell types in the Nui group. Based on the CE scores in the tonsil samples, cell type enrichments were significantly different between the two isolates and between the high and low TVclass samples for four and 12 cell types, respectively (Table 1). Principle component analysis (PCA) plots of the CE estimates across the 51 samples suggested that Isolate and TVclass did not account for large proportions of the variation in the 28 cell types in the Sig group (Figure S2). For the Sig group, the first three principal components (SigPC1, SigPC2, and SigPC3) explained 31.0, 22.0, and 18.4%, respectively, of the variance, while the first three principal components of the Nui group (NuiPC1, NuiPC2, and NuiPC3) explained 55.3, 17.9, and 8.0% of the variance, respectively. The top three cell types explained by each PC are shown in Table S2.

### Sample clustering and differentially expressed genes (DEGs) based on RNA-seq

Hierarchical clustering of the top 200 most variable genes across all samples did not show very clear clustering of samples by any of the factors of interest (see Figure S3), although there was a tendency of some samples to cluster by Isolate, which was consistent with the number of DEGs identified in the model without accounting for CE (Table 2). Differences in cell composition had significant effects on the tonsil transcriptome at 42 dpi based on changes in the numbers of DEGs for Isolate, TVclass, and their interaction, when CE was included in the model, and based on identification of a large number of DEGs for the effect of the fitted PC’s for CE (Table 2 and Table S3). The number of DEGs identified for Isolate was lower when CE was accounted for. TVclass revealed four DEGs when CE was accounted for and zero without CE. For the main effect of WUR, two DEGs were identified in the model with CE and zero DEG without CE. Significant effects of the two-way interactions of WUR with Isolate and TVclass on gene expression were only identified when CE was accounted for. For the effect of sex, the same 24 genes were identified to be DEGs in models without and with CE (Table S3), while correlations of log_2_(Fold Change) (log_2_FC) and q values of these 24 DEGs in the models without and with were 1.00 and 0.94, respectively. Thus, the effect of sex on the tonsil transcriptome did not change substantially when accounting for CE but accounting for CE did impact the effect of other factors.

**Table 2:** Numbers of differentially expressed genes for the factors of Isolate, TVclass, and WUR genotype based on the RNA-seq data with or without accounting for cell enrichment scores

Factor	Without cell enrichment	With cell enrichment	Overlap
Isolate: KS06/NVSL	1074	204	162
TVclass: High/Low	0	4	0
WUR: AB/AA	0	0	0
Isolate*WUR	-^a^	107	-^a^
TVclass*WUR	-^a^	5	-^a^

^a^ This factor was not included in the model

### Effect of isolate based on RNA-seq

For the model without CE (−CE), 1074 DEGs were identified for Isolate, at a q-value threshold of 0.1. In total, 12,244 genes were entered into the Ingenuity Pathway Analysis (IPA) software using pig gene names as gene ID, 10,254 of which were matched with human gene names in IPA. In the canonical pathway analysis, 18 significant pathways related to immunity involved DEGs for Isolate from the model without CE (Figure S4), noting that only pathways that belonged to immune response related categories were included in IPA, i.e. “Cellular Immune Response”, “Cytokine Signaling”, “Humoral Immune Response”, and “Pathogen-Influenced Signaling”. All 18 pathways were predicted to be more activated, or less inhibited, in tonsils of KS06-infected pigs than in tonsils of NVSL-infected pigs (Figure S4). The most significant pathway of these was the “GP6 Signaling Pathway” (Figure S5A), which included 27 Isolate DEGs from the model without CE (Isolate -CE-DEGs).

**Fig. 2:**
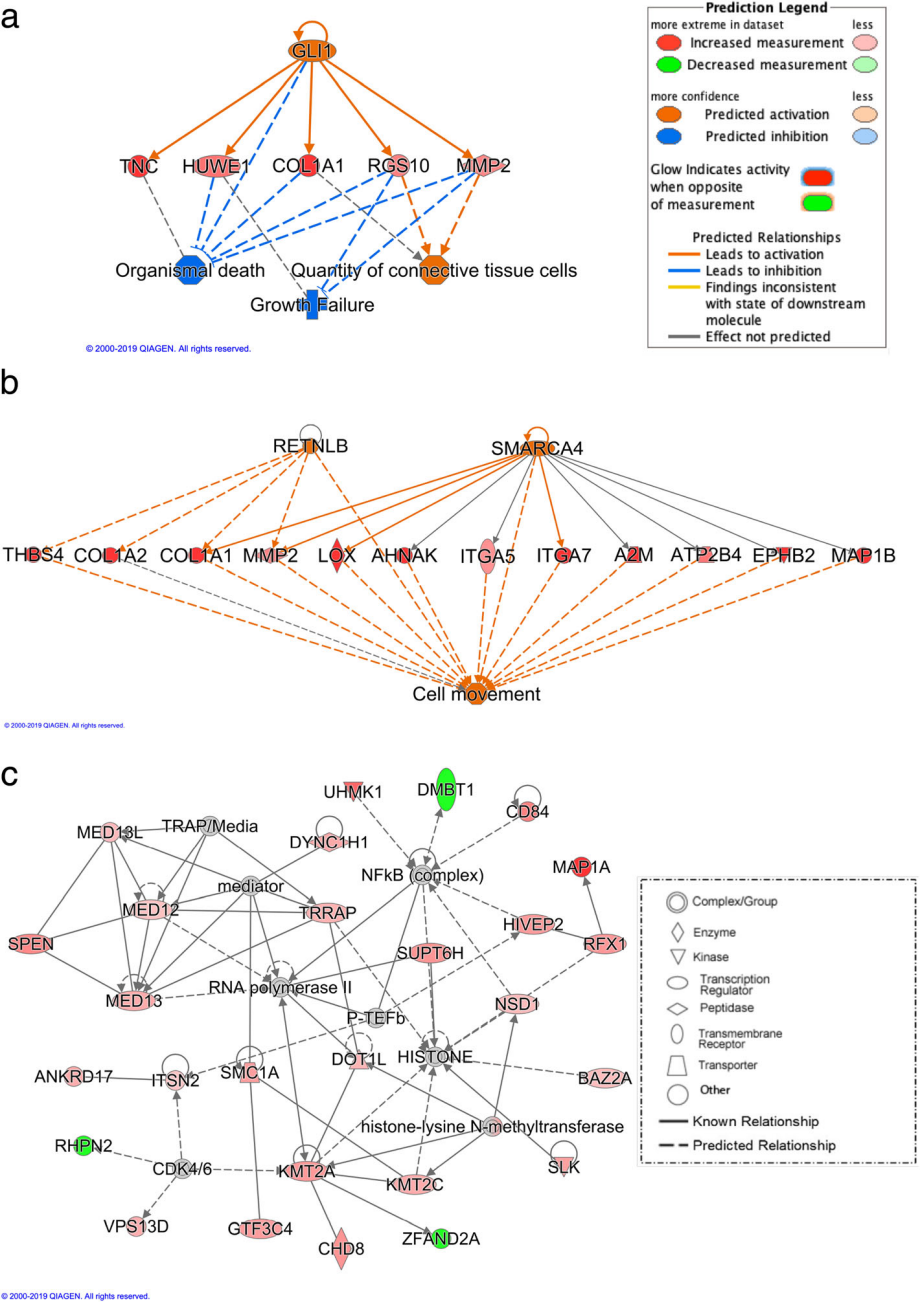
Differences in predicted biological functions and networks in tonsils of pigs infected with the KS06 versus NVSL isolate based on the RNA-seq data by Ingenuity Pathway Analysis. **a** and **b** was used to identify a subset of genes known to be regulated by one and two up-stream regulators, respectively, that included genes with significant differential expression (q-value < 0.1) in tonsils when accounting for cell enrichment. All five and 12 differentially expressed genes were expressed more in tonsils of KS06-infected pigs than of NVSL-infected pigs (intensity of red color indicates magnitude). Orange color (z-score ≥ 2) and blue color (z-score ≤ − 2) indicate activation and inhibition of predicted relationships between these genes, potential up-stream regulators, and potential down-stream functions. Inconclusive genes indicated by grey color. **c** A novel network that includes differentially expressed genes for isolate in tonsils at 42 dpi when accounting for cell enrichment. Dotted lines represent indirect interactions and solid lines are direct interactions. Genes in red (green) were expressed more (less) in tonsils of the KS06-infected pigs than in tonsils of the NVSL-infected pigs. The grey genes were not differentially expressed and white genes were not expressed but predicted by IPA to be involved in the network

When accounting for CE (+CE), only one significant pathway was identified, “GP6 Signaling”, which included 11 DEGs for Isolate (Isolate +CE-DEGs) (Figure S5B). These 11 DEGs were also significant for the model without CE (Figure S5A). Five Isolate +CE-DEGs that were expressed more in tonsils of KS06-infected pigs than in tonsils of NVSL-infected pigs were predicted to decrease “organismal death” and “growth failure” and increase “quantity of connective tissue cells”, regulated by *GLI1* (Fig. 2a). Twelve Isolate +CE-DEGs that were expressed more in tonsils of the KS06-infected pigs were predicted to increase “cell movement”, regulated by *RETNLB* and *SMARCA4* (Fig. 2b). A novel network based on Isolate +CE-DEGs was predicted by IPA to be involved in “cancer”, “hematological disease”, and “immunological disease” (Fig. 2c). The Isolate +CE-DEGs (colored in red in Fig. 2c) in this network were more highly expressed in tonsils of pigs infected with KS06 compared to NVSL, except for *DMBT1*, *RHPN2*, and *ZFAND2A* (colored in green in Fig. 2c). The proteins that were predicted to interact with these DEGs were “NFκB (complex)”, “HISTONE”, “RNA polymerase II”, and “histone-lysine N-methyltransferase” (Fig. 2c).

### Effect of tonsil viral level and WUR genotype based on RNA-seq

For WUR genotype, two DEGs (*EHPA4* and *TENM4*, q = 0.1) were identified in the RNA-seq data using the model without CE, while two different DEGs (*EPHA4* and *DUOX2*, q = 0.1) were identified based on the model with CE. For TVclass, no DEGs were identified based on the model without CE but four DEGs (*FOSB*, *TSPAN10*, *ENSSSCG00000027998,* and a novel gene *XLOC_016982*) were identified based on the model with CE. Except for *TSPAN10*, the other three DEGs had lower expression in the High- than in the Low-TVclass pig tonsils. Although not significant, the expression level of the *GBP5* gene was numerically higher in tonsils of pigs with the AB versus the AA WUR genotype in models with and without CE (q = 0.45 and 0.94), consistent with Koltes et al. [13]. The guanylate binding protein 1 (*GBP1*) gene was not DEG based on WUR genotype, TVclass, or Isolate, either with or without CE in the model.

In total, 107 genes were significant for the interaction between Isolate and WUR in the model with CE (Isolate*WUR + CE-DEGs) (Table 2). Of these, 74 + CE-DEGs had significantly higher levels of expression and three had lower levels of expression for AB versus AA genotype pigs at WUR when infected with KS06 (Table 3). No +CE-DEGs were identified for WUR genotype among the NVSL-infected pigs (Table 3). Based on IPA, only the “Neuroprotective Role of THOP1 in Alzheimer’s Disease” pathway was significant for the effect of WUR genotype within KS06-infected pigs. This pathway included four Isolate*WUR + CE-DEGs (*KLK10*, *KLK12*, *TMPRSS11D*, and *TMPRSS11E*), which were all significantly expressed more in AB than in AA genotype pigs when infected with KS06. For the interaction between TVclass and WUR, five DEGs were identified (Tables 2 and 3). *LOXL4* was significantly expressed more in pigs with the AB than the AA genotype among Low-TVclass pigs (AB_Low > AA_Low). *GRHL3*, *AHNAK*, and *ARHGAP32* had significantly higher expression levels in pigs with the AB than the AA genotype among High-TVclass pigs (AB_High > AA_High), and *ENSSSCG00000027998* was significantly expressed less in AB_High pigs than in AA_ High pigs.

**Table 3:** Comparison of the number of genes that were differentially expressed based on the RNA-seq and NanoString data for the effect of WUR genotype within Isolate and TVclass

	RNA-seq^a^		NanoString^a^	
	log_2_FC > 0^b^	log_2_FC < 0^c^	log_2_FC > 0^b^	log_2_FC < 0^c^
KS06 (AB vs AA)	74	3	14	37
NVSL (AB vs AA)	0	0	26	12
Low-TVclass (AB vs AA)	1	0	14	7
High-TVclass (AB vs AA)	3	1	7	29

^a^ The numbers of differentially expressed genes (q < 0.1) for WUR genotypes within Isolate or TVclass, based on analysis of the RNA-seq and NanoString data, using the model with cell composition

^b^ Gene expression AB>AA within Isolate or TVclass

^c^ Gene expression AB<AA within Isolate or TVclass

### DEGs based on NanoString and their biological functions

**Fig. 3:**
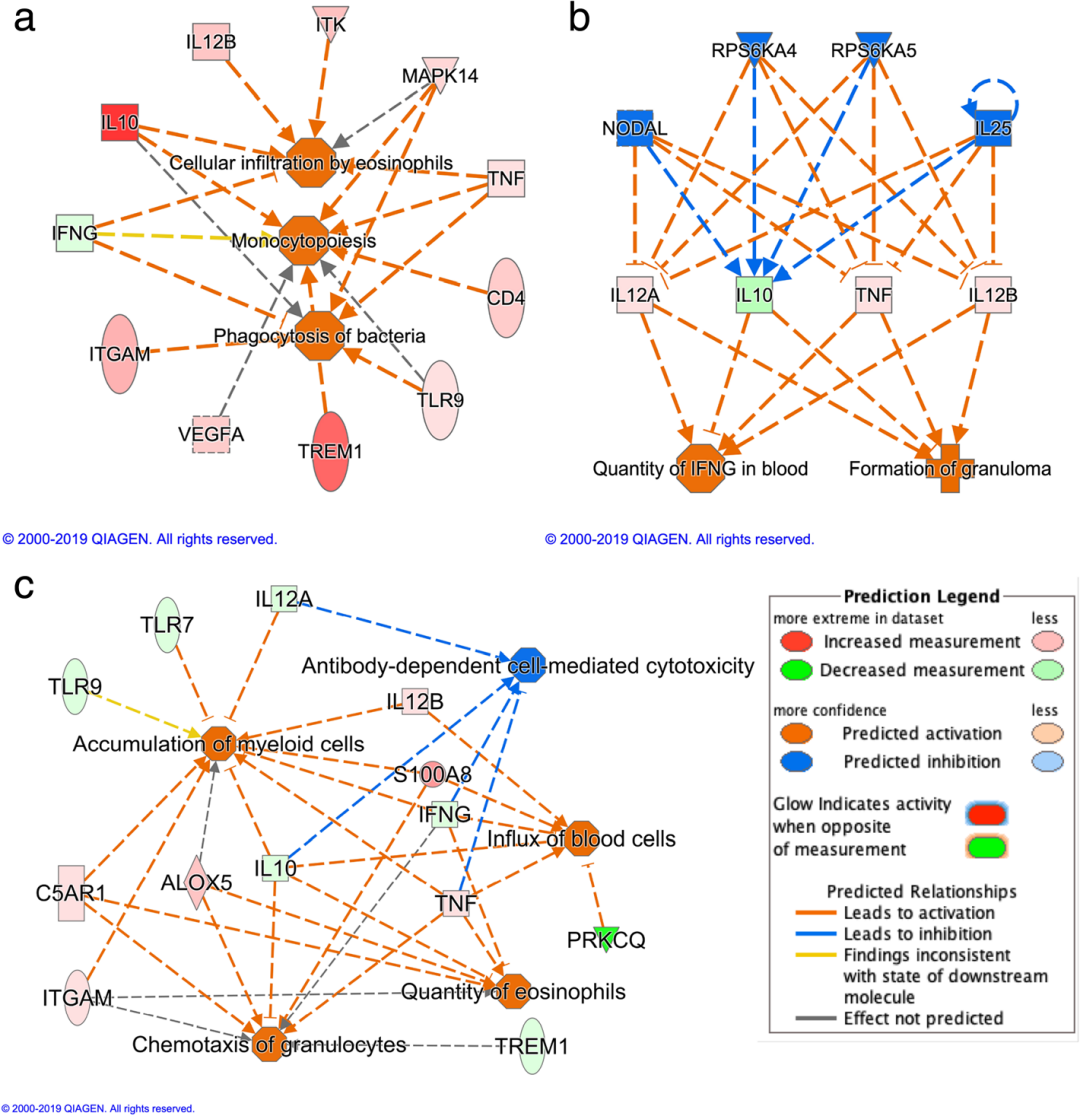
Effects of PRRSV isolate, TVclass, and WUR on gene expression in tonsil based on the NanoString data when accounting for cell enrichment, as predicted by Ingenuity Pathway analysis. **a** The three significant biological functions in orange were predicted to be more activated, or less inhibited, in tonsils of KS06-infected pigs compared to NVSL-infected pigs (magnitude indicated by intensity of red or green). **b** Regulators in blue were predicted to indirectly regulate (dashed line) their downstream differentially expressed genes for TVclass High/Low in red (expressed more in High TVclass) or green (expressed less in High TVclass), which were predicted to have effects on activating “formation of granuloma” or increasing “Quantity of IFNG in blood” (in orange) in tonsils of High-TVclass pigs compared to Low-TVclass pigs. **c** Differentially expressed genes for WUR genotype have effects on five biological functions (orange/blue represents activation/inhibition of the functions). The genes in red/green were expressed more/less in AB pigs than in AA pigs (magnitude indicated by intensity of red/green). The dashed lines represent indirect interactions; the blunt arrow indicates negative regulation and “➔” indicates positive regulation. The synonym of “Antibody-dependent cell-mediated cytotoxicity” is regulation of natural killer cell mediated cytotoxicity

Based on the tonsil NanoString data for the 230 immune related genes, the number of identified DEGs with and without considering CE are shown in Table 4. CE were the same as those used for analysis of the RNA-seq data. Based on IPA, the most significant pathway for isolate for the model without CE was “iCOS-iCOSL Signaling in T helper cells”, which was predicted to be more activated, or less inhibited, in KS06- compared to NVSL-infected pigs (Figure S6A). The Isolate -CE-DEGs also played roles in increasing “transcription of RNA”, “cell movement”, and “quantity of T lymphocytes” in tonsils of KS06- compared to NVSL-infected pigs, which is consistent with the larger CE scores of immune cells in the tonsils of KS06-infected pigs. Expression of the *GBP5* gene was significantly associated with the effect of isolate and was expressed more in KS06- than in NVSL-infected pigs, both with (q = 0.0002) and without (q = 0.0012) CE in the model. Without accounting for CE, the *GBP5* gene was predicted to be more activated, or less inhibited, in KS06- compared to NVSL-infected pigs (*p* = 0.009 and z = 1.38, Figure S7). When accounting for CE, biological functions associated with “activating phagocytosis of bacteria”, “cellular infiltration by eosinophils”, and “monocytopoiesis” involved Isolate +CE-DEGs based on the NanoString data and were more activated, or less inhibited, in tonsils of KS06-infected pigs (Fig. 3a). No pathway was, however, significant for these DEGs based on Fisher’s Exact Test, using a *p*-value < 0.05 and absolute z-score > 2.

**Table 4:** Numbers of differentially expressed genes for the factors of Isolate, TVclass, and WUR genotype based on the NanoString data with or without accounting for cell enrichments

Factor and contrast	Without cell enrichment		With cell enrichment		Overlap of differentially expressed genes
	log_2_FC < 0^a^	log_2_FC > 0^b^	log_2_FC < 0^a^	log_2_FC > 0^b^	
Isolate: KS06 vs NVSL	17	74	9	35	26
TVclass: High vs Low	16	36	13	28	9
WUR: AB vs AA	0	0	21	14	0
(High_AB_-High_AA_) vs (Low_AB_-Low_AA_)	-^c^	-^c^	6	36	-^c^
(KS06_AB_-KS06_AA_) vs (NVSL_AB_-NVSL_AA_)	-^c^	-^c^	64	20	-^c^

^a^ Gene expression of KS06 > NVSL, or Low>High, or AB>AA, or (High_AB_-High_AA_) > (Low_AB_-Low_AA_), or (KS06_AB_-KS06_AA_) > (NVSL_AB_-NVSL_AA_)

^b^ Gene expression of KS06 < NVSL, or Low<High, or AB<AA, or (High_AB_-High_AA) < (Low_AB_-Low_AA_), or (KS06_AB_-KS06_AA_) < (NVSL_AB_-NVSL_AA_)

^c^ This factor was not included in the model

For DEGs for TVclass in the model without CE for the NanoString data, significant pathways were “Interferon Signaling” (Figure S6B) and “Retinoic acid Mediated Apoptosis Signaling”. Both these pathways were predicted to be more activated, or less inhibited, in the High-TVclass pigs, and all DEGs involved in these pathways were expressed at a higher level in the High-TVclass pigs compared to the Low-TVclass pigs. In the model with CE, the pathway “UVC-Induced MAPK Signaling” was significant for TVclass and included four DEGs, *FOS*, *MAPK8*, *PRKCQ*, and *RRAS*, which were all expressed less in tonsils of High-TVclass pigs than in tonsils of Low-TVclass pigs. In addition, *IL12A/B*, *TNF*, and *IL10*, which are potentially regulated by *NODAL*, *RPS6KA4/5*, and *IL25*, had effects on increasing “quantity of IFNG in blood” and activated “formation of granuloma” more, or inhibited it less in High-TVclass pigs compared to Low-TVclass pigs (Fig. 3b).

For WUR genotype, we only identified DEGs in the model with CE based on the NanoString data. There was, however, no significant pathway in IPA for the effect of WUR genotype. However, predicted activation states of “accumulation of myeloid cells”, of “influx of blood cells”, and of “chemotaxis of granulocytes”, and the predicted inhibition state of “antibody-dependent cell-mediated cytotoxicity” were significantly higher in AB compared to AA pigs (Fig. 3c). Moreover, when accounting for CE, expression of the *GBP5* gene was significantly higher in tonsils of AB versus AA pigs, as expected. Although not significant (q = 0.22), the same trends for *GBP5* expression were found when not accounting for CE.

In the model with CE, the interactions of Isolate and of TVclass with WUR were significant for the expression of 84 and 42 genes, respectively (Table 4). For the WUR effect within KS06-infected pigs, only one significant immune response pathway, “IL-8 signaling”, was more activated, or less inhibited, in AB than in AA pigs. In this pathway, five Isolate*WUR DEGs (*IKBKE*, *ITGAM*, *PIK3CG*, *RRAS*, and *VEGFB*) were expressed more in AB than in AA pigs when infected with KS06 (AB_KS06 > AA_KS06), and one Isolate*WUR DEG, *TLR9*, was expressed less in AB_KS06 than in AA_KS06 pigs. No significant pathway was identified for the effect of WUR genotype when pigs were infected with NVSL. For the effects of WUR genotype, only the “interferon signaling” pathway was significantly less activated, or more inhibited, in AB than in AA pigs from the High-TVclass. Three TVclass*WUR DEGs (*IFNG*, *STAT1,* and *TAP1*) were involved in this pathway and they were all expressed less in the AB_High than in the AA_High pigs. For the WUR effect among Low-TVclass pigs, only the “ceramide signaling” pathway was significantly more activated, or less inhibited, in AB than in AA pigs. In this pathway, three TVclass*WUR DEGs (*FOS*, *RRAS*, and *TNF*) were expressed more in AB_Low than in AA_Low pigs, and one TVclass*WUR DEG, *TLR9*, was expressed less in AB_Low than in AA_Low pigs. For expression of *GBP5*, the interaction between Isolate and WUR was significant (q = 0.04) but the interaction of TVclass and WUR was not (q = 0.19).

### Comparison between RNA-seq and NanoString

Of the 230 genes evaluated by NanoString, 203 genes were also detected as expressed in the RNA-seq data. The correlation of the normalized counts obtained from RNA-seq and NanoString for each of these 203 genes ranged from − 0.52 to 0.70, with a mean ± SE of 0.19 ± 0.01. For the *GBP5* gene, the correlation was 0.59. Among these 203 genes, 12 of the 18 Isolate -CE-DEGs based on RNA-seq were also Isolate -CE-DEGs based on the NanoString data (Table 5). These 12 overlapping DEGs had the same trend of expression differences between KS06- and NVSL-infected pigs (Table 6). *S100A8*, the only Isolate*WUR + CE-DEG among these 203 genes based on the RNA-seq data (log_2_FC(AB_KS06/AA_KS06) = 2.04, q = 0.014) was also significant for Isolate*WUR + CE based on the NanoString data (log_2_FC(AB_KS06/AA_KS06) = 2.29, q = 0.00003).

**Table 5:** Numbers of differentially expressed genes for the factors of Isolate, TVclass, and WUR genotype among the 203 genes that were common to the NanoString and RNA-seq analyses

Factor	Without cell enrichments			With cell enrichments		
	RNA-seq	NanoString	Overlap	RNA-seq	NanoString	Overlap
Isolate	18	83	12	2	33	0
TVclass	0	41	0	0	30	0
WUR	0	0	0	0	25	0
Isolate*WUR	-^a^	-^a^	-^a^	1	69	1
TVclass*WUR	-^a^	-^a^	-^a^	0	32	0

^a^ This factor was not included in the model

**Table 6:** Overlapping differentially expressed genes for the effect of Isolate between the NanoString and RNA-seq data analyses without accounting for cell enrichments

Gene name	NanoString data		RNA-seq data	
	log_2_FC^a^	q-value	log_2_FC	q-value
*CDC25B*	0.42	3.96E-03	0.48	0.03
*ETS1*	0.28	0.08	0.45	0.05
*FN1*	0.46	2.83E-04	0.43	0.01
*IFIT2*	0.25	0.01	0.35	0.04
*IKBKE*	0.25	0.02	0.35	0.02
*ITK*	0.29	0.01	0.31	0.04
*JAK3*	0.22	0.02	0.29	0.07
*LAT*	0.37	1.43E-05	0.29	0.08
*LCP2*	0.29	2.65E-06	0.25	0.03
*LYZ*	0.36	2.43E-04	0.24	0.07
*PIK3CG*	−1.28	1.99E-03	−1.08	0.04
*TYK2*	−0.59	0.04	−1.49	0.01

^a^ Log fold change of KS06/NVSL

**Fig. 4:**
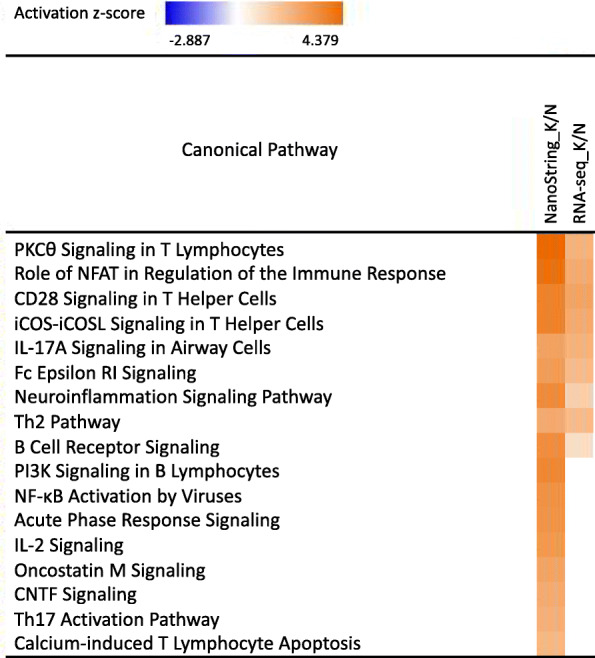
Comparison of immune response related pathways in tonsils at 42 days post infection between the NanoString and RNA-seq data. Comparison of the immune response related pathways that include differentially expressed genes for pigs infected with the KS06 versus the NVSL PRRSV isolate (K/N) based on the model without cell enrichments. The expression values of genes with a false discovery rate < 0.1 were used to calculate -log(*p*-values) and z-scores for each pathway. Orange color (z-score ≥ 2) and blue color (z-score ≤ − 2) indicate activation and inhibition of predicted pathways, respectively. The more intense these colors, the higher the absolute z-scores

In the model without CE, only the effect of isolate showed more than one DEG that overlapped between the RNA-seq and the NanoString data (Table 5). As a result, the comparison of DEG identified using RNA-seq and NanoString in terms of biological function was only possible for this effect. In Fig. 4, the top nine immune response related pathways were predicted to be significantly more activated, or less inhibited, in KS06- compared to NVSL-infected pigs based on both the RNA-seq and the NanoString data. The other eight pathways were only significant based on the NanoString data, which may be due to the limited number of DEGs among the 203 genes based on the RNA-seq data (*n* = 12).

## Discussion

In this study, samples and data from the PHGC experimental infection trials of nursery pigs with PRRSV were used to study viral persistence based on the transcriptome of tonsils at 42 dpi. The PHGC trials were designed to identify genetic variation in host response to PRRSV infection with a specific strain and, therefore, did not include a control group without infection. As a result, we could only compare gene expression differences between pigs with high and low tonsil viral levels and between the two isolates (noting that the effect of isolate was confounded with the effect of trial). Because we did not have baseline gene expression levels in tonsil from uninfected pigs of the same age, we could not determine whether the expression of a gene was up- or down-regulated, or activated or inhibited.

The unique mapping rate of the tonsil RNA-seq data was relatively low (36.7% across samples) and the multiple mapping rate was high (60.9%). This may result from apparent degradation of the tonsil RNA samples, based on the relatively low RIN scores. One possible cause for the low RNA quality may be the time between euthanasia and necropsy (up to one hour), plus additional time to collect tonsil tissue because tonsils are not readily accessible. Within a trial, all pigs were, however, euthanized and necropsied on the same day. We did try to select tonsil RNA samples with higher RIN scores but many had RIN scores less than 5.0.

Differences in host response to infection could also be reflected in differences in tonsil size but this was not recorded. Tonsils are aggregates of lymph nodules and diffuse lymphoid tissues [28], which makes it difficult to remove them completely for weighing. Instead, our gene expression data were based upon a standard sample size. Differences in tonsil size can, however, result from differences in gene expression levels between treatments or factors that may play roles in cell movement, inflammation, immune cell proliferation, or apoptosis. This is consistent with our results of the biological functions of the DEGs, such as “cell movement”, “cellular infiltration by eosinophils”, “monocytopoiesis”, “formation of granuloma”, “accumulation of myeloid cells”, “chemotaxis of granulocytes”, “influx of blood cells”, and “organismal death”.

For TVclass, cause and effect relationships between tonsil viral level and gene expression in tonsils could not be determined. Unlike Isolate or WUR genotype, TVclass was not conducted or pre-selected or assigned before 42 dpi but was classified based on the tonsil viral level at 42 dpi. Therefore, for TVclass, we have two hypotheses: (1) tonsil virus level is the result of differences in immune response, or (2) immune response is the result of the level of tonsil viral load. Because our results showed that pigs with High-TVclass were enriched for immune cells, a high level of virus in tonsil may be the cause of the host’s anti-viral immune response.

Because we did not measure cell composition directly, cell enrichment was determined based on the RNA-seq data using the xCell software. Although the gene signatures for each cell type used by xCell were based on human sequencing data, we believe the results are relevant to pigs. The same holds for the biological function results from IPA, which are mostly based on information from human and mouse literature. Pigs have substantial similarities to humans in anatomy, genome, and physiology and, thus, xCell and IPA are valuable tools to identify significant enrichment of cell types and biological functions of DEGs associated with a treatment or factor of interest. Despite these limitations, we were able to identify several mechanisms associated with PRRSV persistence, which will be discussed in further detail below. These are the first transcriptome data available on tonsils of pigs infected with PRRSV. Therefore, despite the study’s limitations, it is an important contribution to understanding the tonsil transcriptome in nursery pigs infected with PRRSV.

### PRRSV persistence in tonsils and blood

Of the 337 pigs, 168 had a non-zero tonsil viral level, although they had non-detectable levels of serum viremia at 42 dpi (Fig. 1b), representing 80.9% of the NVSL-infected pigs and 22.8% of the KS06-infected pigs. This indicates that absence of serum viremia does not imply clearance of the virus from the body and that the virus can persist much longer in tonsil. This is consistent with previous findings that viral dsRNA persisted in multiple lymphoid tissues [20], although no virus was detected in serum of PRRSV-infected pigs at 52 dpi, thereby escaping antiviral immune response. Therefore, we expected to see prolonged effects of infection on gene expression in tonsil. We found that the 180 NVSL-infected pigs had significantly higher tonsil viral levels (*p* = 0.02) and lower serum viremia (*p* = 0.01) than the 157 KS06-infected pigs, although this difference was not significant for the 53 samples selected for RNA-seq (results not shown). However, Hess et al. [21], using data from the same two trials as used here, along with three additional PHGC trials from other genetic sources, found the difference of tonsil viral levels between NVSL- (*n* = 480) and KS06- (*n* = 302) infected pigs not to be significant. This suggests that the effect of PRRSV isolate on tonsil viral levels at 42 dpi may differ between sources of genetics. For the five trials analyzed by Hess et al. [16], they found that KS06 was more persistent in serum than NVSL, as more KS06-infected pigs had log_10_ serum viremia greater than one at 42 dpi, which is consistent with results from our study on a subset of these data. This indicates that the NVSL strain may be better at avoiding detection and persisting in tonsils by weakening immune responses in tonsils than the KS06 strain. Although we identified a positive relationship between tonsil viral level and serum viremia at 42 dpi, similar to Hess et al. [21], serum viremia was not a good predictor of tonsil viral level at 42 dpi and is, therefore, of limited use in predicting PRRSV persistence.

### Effects of PRRSV isolates, TVclass, and WUR genotype on cell composition

Tonsil tissue consists of multiple cell types, in particular immune cells, including T cells, B cells, macrophages, and DC, which provide, locally and systemically, innate, cellular, and humoral immunity [28]. The factors of interest in this study, i.e. Isolate, TVclass, and WUR genotype, can have effects on cell composition in tonsils, which could lead to identification of DEGs for Isolate, TVclass, or WUR genotype when not accounted for because cell types can differ in gene expression. Because we did not directly measure cell composition of the tonsil samples, we used the xCell software [27] to estimate CE for each sample based on the tonsil transcriptome data. The xCell software uses gene expression signatures for human cell types and may, therefore, not accurately estimate cell type proportions in pig tonsils. Nevertheless, we expect that the PCs we derived from the 62 predicted cell type enrichments do capture general differences in cell composition between samples.

Cell types that were significantly different between factors of interest are listed in Table 1 for the purpose of evaluating effects of the factors of interest on xCell enrichment scores for immune cell types, with *p*-values that were not adjusted for multiple testing. Based on PCA conducted within the Sig and Nui groups, the top 6 and 5 PC’s in the Sig and Nui groups explained over 90% of the variance within each group. Based on this, the number of independent tests conducted was estimated to be 6 + 5 = 11, which, when used for Bonferroni correction, results in an adjusted threshold for significance of 0.1/11 = 0.009. Based on the xCell results (Table 1), only three cell types had significantly larger enrichment scores in the High-TVclass pigs than in the Low-TVclass pigs (q < 0.009): class-switched memory B cells, CD8+ central memory T cells, and basophils. This indicates that pigs with high tonsil viral levels may recruit more immune cells, especially B cells and T cells, to help clear persistent virus from the tonsil. Basophils are involved in anti-parasite immunity and allergic responses. Basophils may also have some unknown function to enhance anti-viral immunity. Although none of the other cell types had significant effects (Table 1), there was a trend towards higher CE scores for specific immune cells for KS06- compared to NVSL-infected pigs and for pigs with the AA versus the AB genotype at WUR. This suggests that the KS06 strain may stimulate a stronger immune response in tonsil at 42 dpi than the NVSL strain, and that AB pigs may not need to recruit as many immune cells in tonsils at 42 dpi to clear the virus as AA pigs.

Our dual use of the RNA-seq data to correct for cell enrichment, i.e. first using the RNA-seq data to predict CE and then fitting the PC’s of the resulting CE estimates in the model to identify DEGs independent of CE, assumes that the expression of genes that are used as gene signatures for a specific cell type is not affected by the factors of interest (Isolate, TVclass, and WUR genotype) [27]. However, because xCell uses more than three gene signatures for each cell type [27], the impact of violation of this assumption is expected to be limited.

### Comparison of RNA-seq and NanoString

We evaluated gene expression using two platforms on the same set of 50 samples, RNA-seq and NanoString. The 12 DEGs that overlapped between the NanoString and RNA-seq results displayed the same trend of fold change (Table 6). Nine of 17 immune response related pathways of the Isolate -CE-DEGs were predicted to be more activated, or less inhibited, in the KS06- compared to the NVSL-infected pigs, based on both types of data (Fig. 4), showing the consistency of these two platforms. However, the correlation of the normalized read counts obtained based on the NanoString and RNA-seq data for the 203 common genes was low, averaging 0.19 and ranging from − 0.52 to + 0.70. These low correlations may be the result of the relatively low RIN scores of the RNA samples, potentially due to RNA degradation during and after sample collection. In RNA-seq, sequencing reads are generated from whole transcripts and, as a result, the RIN score may have an effect on the quality of reads, which was confirmed by the significant effect of RIN on the observed read counts of genes based on the RNA-seq data. To account for the effects of RNA quality in the RNA-seq analyses, we used two weights in the statistical analyses: one that accounts for heterogeneity of sample level variance, which can be due to RNA quality, and an observation level weight that considers the mean-variance relationship of read counts for each gene [29]. Unlike RNA-seq, NanoString just requires ~ 150 bp sequence from each transcript and requires no conversion of RNA to cDNA but identifies the RNA molecules directly. This is expected to make NanoString less sensitive to RNA quality. In contrast to RNA-seq, NanoString also does not encounter the issue of multiple-mapping reads in the alignment process because a molecular barcode is designed specifically for each gene. This also makes NanoString better able to detect low-abundance transcripts than RNA-seq, which depends on read depth to obtain reliable detection [30]. Therefore, we can put more confidence in the NanoString results than in the RNA-seq results for the 203 common genes.

### Effects of PRRSV isolate

Analysis of results obtained from the NanoString data for the effect of isolate based on the model without CE by IPA identified 20 significant immune response related pathways. The “iCOS-iCOSL signaling in T helper cells” pathway was the most significant signaling pathway for the effect of isolate and was more activated, or less inhibited, in tonsils of the KS06-infected pigs (Figure S6A). However, none of these 20 pathways were significant when accounting for CE. This indicates that the expression differences that were identified for immune-related genes between the two PRRSV isolates in the model without CE were due to differences in cell composition of tonsil between the two isolates. This is consistent with what we found for the effect of isolate on CE, which showed that tonsils from the KS06-infected pigs were predicted to have more immune cells, triggering stronger immune responses, compared to tonsils from NVSL-infected pigs. This suggests that the NVSL strain may inhibit or evade host immune response and persist in tonsils more effectively than the KS06 strain. This is consistent with Guo et al. [20], who found that down-regulation of genes involved in B cell and T cell receptor signaling pathways, including ICOS and CD40L expression, results in inhibition of clearance of persistently infected cells, which allows viruses to be maintained in the lymph nodes of persistently infected pigs [20].

Based on the RNA-seq results, the “GP6 signaling pathway” was the most significant pathway that involved DEGs for the effect of isolate based on both models with and without CE. These DEGs were expressed more in KS06- than in NVSL-infected pigs, except for *PI3K* in the model without CE (Figure S5). GP6, a membrane glycoprotein that is expressed in platelets and their precursor megakaryocytes, can stimulate platelet activation and thrombus formation. Platelets play an important role, not only in hemostasis and thrombosis, but also in immunity and inflammation. Many immune related receptors and ligands are present on platelets, including all nine Toll-like receptors (TLRs) and scavenger receptors. Platelets may sample the blood environment to help present the virus to other immune cells [31]. Platelet activation induces the acute phase response to infection and produces IL-1β in mice [32]. In addition, platelets can interact with monocytes and leukocytes via platelet P-selectin [33]. Therefore, our results suggest that KS06 may induce a stronger immune response in tonsils through platelet activation than NVSL, regardless of cell composition.

For the Isolate +CE-DEGs based on RNA-seq, the top novel network predicted by IPA was related to three disease-related functions: “cancer”, “hematological disease”, and “immunological disease” (Fig. 2c). Of the 27 Isolate +CE-DEGs in this novel network, 24 were expressed more in the tonsils of KS06- than of NVSL-infected pigs. These genes were predicted to interact with NF-kΒ, HISTONE, and RNA polymerase II, suggesting that KS06 may cause a stronger immune response by promoting transcription of genes to produce important immune proteins, such as antibodies or cytokines. This is consistent with the IPA regulator prediction that the Isolate +CE-DEGs that were expressed more in the tonsils of KS06-infected pigs, activate cell movement and have less growth failure and organismal death (Fig. 2a and b). This suggests that KS06 infection may induce more immune cell movement to clear the virus from tonsils or to relieve the resulting cell damage.

In summary, our findings suggest that at 42 dpi in tonsils, in order to clear the virus, the KS06 strain stimulates more cell movement and immune cells such as B cells and T cells, greater activation of the iCOS-iCOSL signaling pathway, including NF-kΒ activation in T helper cells through interaction with B cells, and greater platelet activation through the “GP6 signaling pathway”. This suggests that, compared to NVSL, the KS06 strain may have less of an ability to inhibit or evade host immune response and to persist in tonsils.

### Effects of tonsil viral level

Our results suggest that, at 42 dpi, High-TVclass pigs may induce greater aggregation of immune cells in tonsils (Table 1) to trigger a stronger immune response than Low-TVclass pigs. When accounting for CE, the NanoString results identified stronger innate immune responses in High-TVclass than in Low-TVclass pigs, with more IFNG in blood and activation of granuloma formation (Fig. 3b). Based on the RNA-seq data, when accounting for CE, the DEG for TVclass that were not evaluated using NanoString, also had functions to induce stronger immunity in High-TVclass pigs. For example, the *TSPAN10* gene had significantly higher expression in High-TVclass than in Low-TVclass pigs. The *TSPAN10* gene has been implicated in Notch activation by promoting *ADAM10,* which plays an essential role as a “molecular scissor” in ligand-dependent cleavage of the Notch protein and in control of cell fate decisions [34]. Conditional knockouts of *ADAM10* in mice revealed impaired B-, T-, and myeloid cell development and/or function [35–37]. This suggests that High-TVclass pigs may induce a stronger immune response than Low-TVclass pigs related to B, T, and myeloid cell development and/or function through the Notch signaling pathway in tonsils. Therefore, *TSPAN10* is a potential candidate gene to target for control of PRRSV persistence.

Based on the tonsil RNA-seq data, the *FOSB* gene was not only a TVclass +CE-DEG (expressed more in Low-TVclass pigs when accounting for CE) but also an Isolate +CE-DEG (expressed more in NVSL-infected pigs) and an Isolate*WUR + CE-DEG. In the NanoString data, *FOS* was a TVclass DEG and was expressed more in tonsils of Low-TVclass pigs, with or without accounting for CE, but the *FOSB* gene was not included on the NanoString panel. Both *FOS* and *FOSB* are members of the *FOS* gene family and are activator protein-1 (AP-1) transcription factor subunits. FOS proteins play a role in regulating cell proliferation, differentiation, and transformation. A previous study showed that the *FOSB* gene was up-regulated in alveolar macrophages during the early phase of infection with a European PRRSV strain [38]. However, based on the RNA-seq data from this study, when accounting for CE, the *FOSB* gene was expressed more in tonsils of Low-TVclass than of High-TVclass pigs. These contradictory results may be due to differences between tNorth American and European serotype PRRSV strains, different infection stages, or the difference between tissues examined (tonsils versus alveolar macrophages).

### Effects of WUR genotype on tonsil gene expression

Based on the tonsil RNA-seq data, two DEGs were found for the effect of WUR genotype for both models (with and without CE). The *EPHA4* gene was DEG for both models (q-value equal to 0.1 for both) and was expressed more in AB than in AA pigs. Unfortunately, *EPHA4* was not included on the NanoString panel. Although the low expression level of *GBP5* gene makes it difficult to detect, especially with RNA-seq, it did have significantly higher expression in tonsils of AB than of AA pigs based on the NanoString data in the model with CE. Although not significant, the same directional trend was found for *GBP5* gene based on the RNA-seq data in the model with and without CE (q = 0.45 and 0.94) and in the model without CE based on the NanoString data (q = 0.22). These trends are consistent with results by Koltes et al. [13] for expression in blood following PRRSV infection without accounting for CE. In human studies, Huttlin et al. [39] found that the GBP5 protein interacts with several other proteins in HEK293T cells, including EPHA4, GBP1, and SURF1, as well as with other genes that are currently not in the pig Ensembl database. We found that both *GBP5* and *EPHA4* gene were expressed more in AB than in AA pigs in the model with CE. However, in our RNA-seq data, expression of *SURF1* was not detected in tonsils and *GBP1* was not significantly associated with WUR genotype for either model. The *EPHA4*, *GBP1,* and *SURF1* genes were not included on the NanoString panel. In mouse, Rothgiesser et al. [40] found that both *GBP5* and *EPHA4* were NF-κB-dependent genes. Higher expression of *EPHA4* and *GBP5* in AB than in AA pigs is expected to induce a stronger immune response for AB pigs.

### The effect of WUR genotype differs by isolate

The *S100A8* gene is an important pro-inflammatory mediator that had significantly greater expression in tonsils of AB than of AA pigs when infected with KS06, based on both the NanoString and the RNA-seq data when accounting for CE. Among the NVSL-infected pigs, *S100A8* was not a DEG for WUR based on either the RNA-seq or the NanoString data. Based on the NanoString data, the IL-8 signaling pathway was more activated, or less inhibited, in AB than in AA pigs, when infected with KS06. IL-8 is also known as CXCL8, belongs to the CXC chemokine family, and can bind to CXCR1 and CXCR2 as cell-surface receptors and stimulate exocytosis, chemotaxis, and signal transduction, depending on the cell type [41]. Thus, our results suggest that, when infected with KS06, AB pigs activated a stronger innate immune response in tonsils at 42 dpi than AA pigs. Of the 107 identified Isolate*WUR + CE-DEGs based on the RNA-seq data, 74 (69%) had significantly higher expression in AB than in AA pigs when infected with KS06 but there was no significant difference between AB and AA pigs for these genes when infected with NVSL. This suggests that, at the transcriptome level, WUR genotype, which has an effect on *GBP5* gene expression [13], had a larger effect in the tonsils of KS06-compared to NVSL-infected pigs. Four of the Isolate*WUR + CE-DEGs are serine proteases (*KLK10*, *KLK12*, *TMPRSS11D*, and *TMPRSS11E* based on the RNA-seq data), which play essential roles in inflammatory responses and in innate and adaptive immunity [42]. These genes were expressed more in the tonsils of AB than of AA pigs when infected with KS06 and accounting for CE, but they were not DEGs for WUR in NVLS-infected pigs, which was consistent with the NanoString results. In summary, the AB genotype at WUR was associated with stronger immune response in tonsils at 42 dpi, especially innate immune response, for pigs infected with KS06 but not for pigs infected with NVSL.

### Effects of the interaction between WUR and TVclass

The *FOS* gene, which is a subunit of AP-1 that can stimulate activation of inflammatory genes in the “ceramide signaling” pathway, was significantly more activated in AB than in AA Low-TVclass pigs based on the NanoString results and when accounting for CE, while no significant difference was observed for the High-TVclass pigs, nor based on the RNA-seq data in either TVclass. Based on the tonsil RNA-seq data, *LOXL4* was the only significant DEG for WUR within the Low-TVclass. The LOXL4 protein contains a highly conserved protein module, Scavenger Receptor Cysteine-Rich domain, which plays roles in innate immune response [43]. The *LOXL4* gene can be stimulated by TGF-β1 through the Smad and AP-1 complex, which is composed of JunB/Fra2 and plays a role in vascular extracellular matrix homeostasis [44]. This suggests that the greater expression of *LOXL4* in AB versus AA pigs among the Low-TVclass pigs may result in stronger innate immune response in AB than in AA pigs, but with no effect among High-TVclass pigs. This is consistent with what we found based on the NanoString data.

Based on the NanoString results within the High-TVclass pigs, three TVclass*WUR + CE-DEGs (*IFNG*, *STAT1,* and *TAP1*) had lower expression in AB than in AA pigs, and either activated the “interferon signaling” pathway less, or inhibited this pathway more, in AB than in AA pigs. Based on the NanoString data, all three genes had numerically higher expression in AB than in AA pigs within the Low-TVclass group, but these differences were not significant. Based on the RNA-seq data, *STAT1* and *TAP1*, also had numerically lower expression in AB than in AA pigs within the High-TVclass group but, again, these differences were not significant (q = 0.17 and 0.92), while *IFNG* was not identified in the RNA-seq data. The five TVclass*WUR + CE-DEG identified in the RNA-seq data were not on the NanoString panel.

Based on the RNA-seq data, *GRHL3* was one of five TVclass*WUR + CE-DEGs that had significantly higher expression in AB than in AA pigs within the High-TVclass group, but not within the Low-TVclass group. The *GRHL3* gene was also an Isolate*WUR + CE-DEG and had significantly higher expression in AB than in AA pigs when infected with KS06, but not among NVSL-infected pigs. A previous study showed that the GRHL3 protein decreases expression of mouse miR-21 mature microRNA in differentiated normal human keratinocytes [45]. miR-21 is a negative regulator for *TLR4* to activate NF-κB and decrease IL-10 production by targeting a pro-inflammatory tumor suppressor *PDCD4* [46]. This suggests that *GRHL3* may suppress miR-21 and, subsequently, activate NF-kB and decrease IL-10 production. Based on our results, higher expression of *GRHL3* in AB pigs is predicted to stimulate innate immune responses more in AB than in AA pigs within the High-TVclass group or when infected with KS06, but has no significant effect within the Low-TVclass group or when infected with NVSL.

The *AHNAK* gene, which is highly expressed in CD4+ T cells [47], was another TVclass*WUR + CE-DEG, that had significantly higher expression in AB than in AA pigs within the High-TVclass group, but was not significant within the Low-TVclass group. The *AHNAK* gene plays an important role in T cell calcium signaling triggered by TCR activation [47]. This suggests that the *AHNAK* gene may have an effect on T cell activation, depending on the interaction effect of TVclass and WUR genotype.

The *ARHGAP32* gene also had significantly higher expression in AB than in AA pigs within the High-TVclass group but was not significant within the Low-TVclass group. The ARHGAP32 protein (also known as p200RhoGAP, p250GAP, GC-GAP, Rics, or Grit) plays a role in activating GTPase of the Rac1 and RhoA proteins [48]. The Rac1 protein plays an essential role in regulating NF-κB transcriptional activity in cells of the innate immune system [49]. Activation of RhoA is required for production of pro-inflammatory cytokines mediated by lipopolysaccharide in human monocytes through TLR4 and TLR2 [49]. Thus, *ARHGAP32* may affect the level of innate immune response by increasing GTPase activity, depending on the interaction effect of TVclass and WUR.

In summary, the effect of WUR genotype on the tonsil transcriptome depends on the tonsil viral level and the PRRSV isolate. Further study is needed to validate these interaction effects with WUR genotype.

## Conclusions

Transcriptional profiling in tonsils at 42 dpi revealed an immune response to PRRSV persistence that may have been influenced by intercellular communication with other body tissues through blood. The impact of PRRSV isolates on host immune responses provides insights into the potential means of viral persistence in tonsils. In summary, we found higher immune responses in tonsils of KS06-infected pigs and in pigs with high levels of virus in tonsil, than in tonsils of NVSL-infected pigs and in pigs with low levels of virus in tonsil at 42 dpi. Based on these results, we hypothesize that NVSL can persist in tonsils longer than KS06. Moreover, the presence of more virus in tonsils (high tonsil viral level) induces stronger immune responses, especially innate immune responses. Genes that were significantly impacted by the interaction of WUR genotype with isolate may play a critical role in innate immune response, especially the IL-8 signaling pathway, which indicates that the *GBP5* gene may have different effects on tonsil immune responses during persistence in pigs infected by different PRRSV strains.

This study also highlights the importance of measuring cell composition in heterogeneous tissues. Accounting for cell composition allowed us to more comprehensively understand the mechanisms of PRRSV persistence. Although we used the xCell software, which is designed based on human data, to predict CE scores in porcine tonsils, our data affirmed the importance of cell migration during persistence. Integration of information on novel genes from the Iso-Seq data provided important information on differential expression of these genes in tonsils, which can be potential candidate genes to help control PRRSV persistence. Future studies should examine protein expression levels and validate the estimated cell composition in tonsils. Use of single cell sequencing and gene knockouts would help confirm mechanisms of PRRSV persistence and identify candidate genes to target to decrease PRRSV persistence.

## Methods

### Experimental design, sample collection and PRRSV test

At an average age of 26.4 ± 0.7 days, 184 and 180 Duroc×Landrace/Yorkshire commercial nursery pigs from the same genetic source, were experimentally infected (intramuscularly and intranasally) with 10^5^ (TCID50) of the NVSL or KS06 strains of PRRSV, respectively, in two separate PHGC infection trails (trial PHGC5 with NVSL and trial PHGC14 with KS06) [16]. A total of 27 pigs that died or that were euthanized for humane reasons before 42 dpi were not included. At 42 dpi, all remaining pigs were euthanized, and serum and tonsil samples were collected (to the greatest extent possible, the central portion of the tonsil, at the oral side of the soft palate, was sampled through its entire depth). Animals were humanely euthanized by pentobarbital overdose following the American Veterinary Medical Association guidelines for the euthanasia of animals, and all efforts were made to minimize suffering. Tonsil samples were frozen and stored at -80 °C until analyzed. Tonsil viral level and serum viremia were evaluated by a semi-quantitative PCR assay for PRRSV RNA, as described in Boddicker et al. [14] and Hess et al. [21]. Two assays, the Applied Biosystems AgPath ID NA and EU PRRSV reagents (AB assay), and the Tetracore US and EURO PRRSV Master Mix reagents (Tetracore assay), were used to measure the tonsil viral levels for the NVSL and KS06 trails, respectively, because the cDNA from the KS06 isolate failed to be amplified by the AB primers [21]. Therefore, the tonsil virus levels for NVSL were adjusted to their Tetracore equivalent, as described in Hess et al. [21]. VLTotal in serum was calculated by area under the Wood’s curve from infection until euthanasia at 42 dpi, as a measure that includes both the level of viremia and the extent to which viremia is maintained [16, 50]. Additional details of these trials and assays are presented in Hess et al. [21].

### Sample selection

Total RNA was isolated from aliquots of frozen tonsil samples using RNeasy RNA isolation kit (Qiagen, Germantown, MD, USA) according to the manufacturer’s protocol. RNA concentration was first measured with a NanoDrop ND-1000 UV-vis Spectrophotometer (NanoDrop Technologies Inc., Wilmington, DE, USA). The RIN score of the extracted RNA was determined for each sample using the RNA Nano 6000 Assay kit on an Agilent Bioanalyzer 2100 system (Agilent Technologies, Santa Clara, CA, USA). A total of 53 tonsil samples were selected for gene expression analyses based on high or low PRRSV level in the tonsil (at least one standard deviation from the mean) (High and Low-TVclass) and RIN score of the extracted RNA (Figure S1). Figure 1 illustrates the samples that were selected for RNA-seq; for PHGC5, *n* = 15 NVSL_High, with tonsil viral level at 42 dpi (TV42) = 6.81 ± 0.56 log_10_(viral copies/mg)), *n* = 15 NVSL_Low with TV42 = 3.65 ± 0.42 log_10_(viral copies/mg); for PHGC14, *n* = 12 KS06_High with TV42 = 4.63 ± 0.28 log_10_(viral copies/mg), and *n* = 11 KS06_Low with TV42 = 2.11 ± 0.37 log_10_(viral copies/mg)). The average age of the pigs for the 53 selected samples was 26.5 ± 0.8 days and their gender information is shown in Figure S3. NanoString analyses were performed on these same samples. All pigs were genotyped for the WUR SNP (rs80800372), as part of the 60 K panel for which all PHGC pigs were genotyped (13 AB and 38 AA pigs for RNA-seq, Figure S3).

### RNA-sequencing and preprocessing of RNA-seq reads

For tonsil RNA-seq, library construction and sequencing were performed by the DNA facility at Iowa State University. The cDNA libraries of the selected 53 RNA samples were constructed from total RNA using the Illumina TruSeq® RNA Sample Preparation Kit v2 (Illumina, San Diego, CA, USA) according to manufacturer’ s instructions. The cDNA from all 53 samples was pooled in approximately equimolar amounts after ligating adapters with unique barcodes for each sample and loaded on each of five lanes of an Illumina® HiSeq 3000 flow cell for 100 base paired-end sequencing.

Raw reads from the fastq files obtained from each of the five lanes were checked for quality using FastQC (Version 0.11.3) [51], trimmed using Trimmomatic (Version 0.36) [52], and aligned to build 11.1 of the pig genome using STAR (Version 2.5.3) [53]. To avoid multiple mapping issues for the hemoglobin (HB) genes, *HBB-like* (ENSSSCG00000014727) and one of two similar exon sequences within *HBA* (ENSSSCG00000007978) were masked in the reference genome sequence. For gene annotation, Ensembl pig 11.1 gene annotation was used, combined with annotation for an additional 12,491 novel genes that were identified by Pacific Biosciences Iso-Seq of a cross-bred pig and RNA-seq data of nine pig tissues (brain, hypothalamus, liver, muscle, thymus, pituitary, small intestine, spleen, and diaphragm) [26]. The current version of Ensembl gene annotation (SSC11.1) does not include *GBP5.* Instead, the WUR SNP (rs80800372) and the putative causative *GBP5* intronic SNP (rs340943904) are located in three of nine transcripts that are incorrectly annotated as *GBP1*: ENSSSCT00000065307, ENSSSCT00000060466, and ENSSSCT00000044130 [13]. Thus, the gene annotation file was changed to recognize these three transcripts as *GBP5*. BAM output files from STAR were used to build an index using Samtools (Version 1.3.1.1) [54] and used as inputs for counting reads using htseq-count [55] with the merged Ensembl gene annotation and IsoSeq novel gene annotation as a gtf file. The resulting read count data for each of the five lanes were then combined into one dataset for each tonsil sample. Two samples in the NVSL-low group with low read counts and high proportions of zero read counts (89.1 and 91.2%) were excluded from further analysis, leaving 51 samples.

### NanoString gene expression analysis

All but one (Low TVclass, KS06 Isolate, and AA WUR genotype, marked in red in Fig. 1) of the 51 samples used in RNA-seq were also evaluated using NanoString gene expression technology. RNA preps were diluted with RNA-free water to 25–100 ng/ul. Using a uniquely designed swine immune codeset (nCounter XT CodeSet Gene Expression Assay NanoString Technologies Seattle, WA), hybridization buffer was added to the reporter codeset to create a master mix. An aliquot of the mastermix was mixed with 5 ul of each RNA sample, followed by the capture probe. The samples were placed at 65 °C in a pre-heated thermal cycler for at least 16 h. One sample with 100 ng RNA per lane was loaded and then processed in the nCounter (NanoString Technologies) [31]. The custom-designed CodeSet of 230 genes was selected from genes and pathways associated with porcine blood, lung, lymph node, endometrium, placenta, or macrophage response to infection with PRRSV [56]. These genes are involved in innate and adaptive immune response, as well as apoptosis and mitosis pathways based on IPA and KEGG Pathway Database [56]. Not all genes in a given pathway were selected to avoid over-representing pathways. In addition, some selected genes are involved in multiple pathways. Nine housekeeping genes were included in the CodeSet.

### Gene expression data visualization

For data visualization, hierarchical clustering was performed using R version 3.3.1 [57]. The 51 RNA-seq samples were clustered to identify any obvious outliers and for clustering of samples by factors of interest, including Isolate, TVclass, WUR, sex, and RIN. We calculated the sample distance matrix using the “euclidean” method, applied hierarchical clustering using the “average” agglomeration method of the *hclust* R function, PCA using the *prcomp* R function without scale, and plotted sample dendrograms against the factor colors using the *plotDendroAndColors* R function. The 200 most variant genes were used after upper quartile normalization.

### Cell composition estimation

Tonsils consist of numerous cell types [28], so any DEGs identified could be the result of changes in cell composition. The software xCell [27] was used to estimate the CE of each sample for 64 immune and stromal cell types (Table S1) based on the gene expression levels of signature genes for each cell type. All 51 samples were analyzed simultaneously, using the tonsil RNA-seq data for 6993 genes for which *Sus scrofa* gene names from Ensembl overlapped with the xCell gene list, which includes 10,782 genes with human gene names. The output data, used for subsequent analyses, were the CE scores after transformation of raw cell composition scores to percentages and adjustment using the spill over compensation matrix from xCell [35]. We analyzed the resulting CE scores for each cell type using a linear model with the fixed effects of Isolate, TVclass, WUR, sex, RIN (covariate), age (covariate), and the 2-way and 3-way interactions among Isolate, TVclass, and WUR. The best model for each cell type was selected based on the Bayesian information criterion (BIC) through backward selection using the stepAIC function in R. ANOVA was used to test significance of the effects of the factors or covariates in the best model for each cell type. Based on this, cell types were assigned to a “significant cell type” group (Sig) if at least one of the factors of Isolate, TVclass, WUR, their interactions, sex, or age, was significant (*p* < 0.1) for that cell type; otherwise, it was assigned to the “nuisance cell type” group (Nui). The rationale for this was that effects of cell types in the Sig group were more important to account for in the differentially expression (DE) analysis than the effects of cell types in the Nui group because the former are expected to impact the estimates of effects of factors of interest on DE analysis. However, because cell types in the Nui group may explain part of the random variation in the gene expression data, they are also be important to consider in DE analysis. Considering the limitation of our sample size (*n* = 51), PCA was used for each group of cell types for dimensionality reduction for CE scores for inclusion in the DE analysis.

A linear model with the fixed effects of Isolate, TVclass, WUR, sex, and RIN (covariate), which was the same as the linear model used in the DEG analysis without CE using the tonsil RNA-seq data, was used to determine the effect of factors of interest (Isolate, TVclass, and WUR) on the CE scores for each cell type. The lsmeans function of R was used to estimate differences in least squared means for each cell type to quantify the effects of Isolate, TVclass, and WUR genotype on CE. The Bonferroni correction set the significance cut-off at 0.003 (α/ n, α = 0.1, *n* = 33, the number of the immune cell types in xCell).

### Differential expression analysis of tonsil RNA-seq data

The R language (version 3.3.1) was used for all statistical analyses. After removing transcripts with mean read counts across all 51 samples less than eight and less than four samples with read counts larger than zero, 19,107 genes were determined to be expressed in tonsil at 42 dpi. Of these, 12,243 were annotated in Ensembl pig 11.1, while the rest were novel genes identified by Iso-Seq [26]. This included the *GBP5* gene, which was annotated as being part of the *GBP1* gene in Ensembl pig 11.1.

Differential expression analyses were performed using the linear model pipeline implemented in the *voomWithQualityWeights* function of the R *limma* package [29, 58]. Briefly, read counts were first log-transformed as log_2_[(n + 0.5)*10^6^/(m + 1)], where n is the read count of a given gene in a sample and m is the upper quartile of read counts in the sample across all genes [36]. Then, sample level weights and observational level weights were estimated. Using the combination of these two weights in the linear model for statistical analysis can increase power and reduce false discoveries compared to other methods [29]. In the linear model, covariates and fixed effect factors considered included Isolate, TVclass, WUR, sex, RIN, age, and tonsil viral level. To identify the best model to be used across genes in terms of inclusion of the covariates of age, RIN, and tonsil viral level, the backward variable selection method of Nguyen [59] was applied. This method uses pseudo-covariates to control the expected proportion of selected covariates that are irrelevant for the RNA-seq dataset [59]. Factors not subject to variable selection were sex and the combination of Isolate, TVclass, and WUR. After variable selection, the 2-way and 3-way interactions among Isolate, TVclass, and WUR from the selected model were considered for inclusion in the final model. The same method was used when including CE in the model, by considering the first three principal components of each of the significant and nuisance groups of cell types (SigPC1, SigPC2, SigPC3, NuiPC1, NuiPC2, and NuiPC3) as candidate covariates. Storey’s method [60] with an estimate of the proportion of true null hypotheses using the histogram-based method of Nettleton et al. [61] was applied to control the false discovery rate (FDR) at less than 0.1 for identification of DEG for Isolate, TVclass, and WUR. In order to understand the biological function of DEGs that were significant for the Isolate*WUR interaction, we evaluated contrasts between AB genotype pigs infected with KS06 (AB_KS06) and AA genotype pigs infected with KS06 (AA_KS06) to identify the DEGs for WUR within KS06-infected pigs, and the same for DEGs for WUR within NVSL-infected pigs (AB_NVSL vs AA_NVSL), High-TVclass pigs (AB_High vs AA_High), and Low-TVclass pigs (AB_Low vs AA_High). Each of the comparison can be called as a simple effect of WUR. For these simple effects of WUR, we only considered DEGs that overlapped with DEGs for the interaction effect between WUR and Isolate or TVclass, which identifies genes for which the effect of WUR differs between the two isolates or between pigs with high and low tonsil viral level.

### Statistical analysis of NanoString data

The raw NanoString nCounter data were analyzed using the NanoStringDiff package in R [62]. NanoStringDiff fully utilizes the normalization information provided by the nCounter system, including housekeeping genes, six positive controls, and eight negative controls [62]. The expression of housekeeping genes is expected to be stable across samples, but this is not always the case [62]. Therefore, we checked the expression of the nine housekeeping genes that were included in the NanoString CodeSet in the RNA-seq data of these same samples and removed the five genes that were DEGs for any factors or covariates in the model without CE as housekeeping genes, leaving *HPRT1*, *TBP*, *YWHAZ*, and *SDHA* (for Isolate and/or RIN). Although the latter two genes were DEG in the model with CE, all four genes were maintained as housekeeping genes in our analyses because NanoString analysis requires at least three housekeeping genes.

The model for DE analysis of the normalized NanoString data was the same as the model used for analysis of the RNA-seq data, including Isolate, TVclass, WUR, sex, and RIN, with or without considering PC’s for CE predicted by xCell based on the tonsil RNA-seq data. The simple effect of WUR was also tested. Genes with a q-value less than 0.1 were defined as DEGs.

### Biological function and pathway enrichment analyses

To identify over-represented canonical pathways associated with DEGs, the *p*-values, q-values, and estimates of log_2_FC of all genes for the effects of Isolate, TVclass, and WUR in the model for each gene were uploaded in the IPA software [63]. Because IPA does not recognize pig Ensembl gene ID’s, 12,876 of the 19,106 genes identified in our tonsil RNA-seq data were converted from Ensembl pig gene ID to pig gene names using BioMart in Ensembl, resulting in 11,841 pig gene names identified by “Gene symbol-human (Hugo/HGNC, Entrez Gene)” in IPA. For the NanoString results, log_2_FC values, *p* values, and q values of 228 of the 230 genes that were identified by IPA were entered into IPA.

For IPA, the same settings were used for analysis of results from the RNA-seq and the NanoString data. After setting “Create Core Analysis” and 0.1 as the q-value cutoff, the “User Dataset” and “Direct and Indirect Relationships” were chosen as “Reference Set” and “Relationships to consider”, respectively. In the canonical pathway analysis, the cutoff of Fisher’s Exact Test p-value was set to less than 0.05 and the cutoff for the absolute value of the z-score was set to be greater than 2.0.

### Comparison between RNA-seq and NanoString

In order to make a more reliable comparison between RNA-seq and NanoString data based on the 50 overlapping samples, we extracted the data from the 203 genes from the tonsil RNA-seq data that overlapped with the 230 genes in the NanoString data. Of the 27 NanoString genes that were not in the RNA-seq expressed data, 24 had low read counts in RNA-seq and were filtered out before DE analysis. The other three, *OAS1*, *C3AR1*, and *IFNA*, did not have corresponding Ensembl ID’s in the Ensembl database. Using the data on the 203 genes, we first compared the correlation of the normalized RNA-seq and NanoString read count data, separately for each gene. We used the same normalized methods as described above for DE analyses. For the RNA-seq data, we log-transformed read counts for DEG analysis using the *voomWithQualityWeights* method in the R *limma* package. For the NanoString data, we transformed read counts as log_2_(n + 0.5), where n is the normalized count of a given gene extracted from NanoStringDiff. Second, we checked the DEGs among these 203 genes that overlapped between RNA-seq and NanoString for each factor (Isolate, TVclass, and WUR). Third, the biological functions of DEGs were compared based on analysis of the RNA-seq versus NanoString data for these 203 genes by IPA.

## Supplementary Information

**Additional file 1: Figure S1.** The relationship between RIN score and tonsil viral level in pigs infected with NVSL (A) and KS06 (B). The samples associated with selected samples for RNA-seq (High-TVclass in red; Low-TVclass in green) and unselected samples (in blue) were converted to a color representation. **Figure S2.** Plots of each two of top three principle components (PC) for the 28 cell types that were significantly(*p*<0.1) affected by at least one of Isolate, TVclass, WUR genotype, their interactions, sex, or age. The cell compositions in tonsils were predicted based on the tonsil RNA-seq data using the xCell software. Shapes indicate samples from NVSL or KS06-infected pigs. Colors indicate samples with high or low tonsil viral level. PC’s 1, 2, and 3 explained 31.0, 22.0, and 18.4%, respectively, of the variance in cell composition of the 28 cell types. **Figure S3.** Hierarchical clustering of the 200 most variant genes based on upper quartile normalized read counts. Fifty-one RNA-seq samples were clustered to identify any obvious outliers and potential clustering of samples by factors of interest, using euclidean method to calculate the sample distance matrix, and the average agglomeration method to apply hierarchical clustering. The samples associated with the factors of Isolate (white=NVSL; red=KS06), TVclass (white=High; red=Low), WUR genotype (white=AA; red=AB), sex (white=Female, red=Male), and RIN (low ➔ high = white ➔ red) were converted to a color representation. **Figure S4.** Significant canonical pathways related to immune response that include differentially expressed genes for isolate in tonsils at 42 days post infection when not accounting for cell enrichments based on the RNA-seq data. The expression values of genes with a false discovery rate <0.1 were used to calculate the z-scores and -log(*p*-values) for each pathway. Pathways shown in this figure have a -log(*p*-value)>1.3 and an absolute z-score > 2. The height of each bar corresponds to the -log(*p*-value). The orange color (z-score≥2) and blue color (z-score ≤-2) indicate activation and inhibition of predicted pathways, respectively. The intensity of these colors indicates the magnitude of the absolute z-score. **Figure S5.** Effect of PRRSV isolate on the pig tonsil RNA-seq transcriptome at 42 days post infection. Only one significant pathway, “GP6 Signaling Pathway”, was identified in the models without (A) and with (B) cell enrichments, which included the same 11 differentially expressed genes for both models. The genes that were expressed more (less) in tonsils of KS06-infected pigs than of NVSL-infected pigs are in red (green). The intensity of shading is proportional to the fold difference in gene expression. The solid lines between genes represent known direct interactions and dashed lines represent indirect interactions; the blunt arrow indicates negative regulation and “➔“ indicates positive regulation. **Figure S6.** Effect of PRRSV isolate and TVclass on the expression of 230 immune related genes in tonsil at 42 days post infection based on the NanoString data without accounting for cell enrichments. A) The most significant signaling pathway, “iCOS-iCOSL Signaling in T Helper Cells”, that included differentially expressed genes for KS06/NVSL Isolate. Twenty-two genes were expressed more in tonsils of KS06-infected pigs than of NVSL-infected pigs (magnitude indicated by intensity of red color). Only one gene, *PRKCQ*, was expressed less in tonsils of KS06-infected pigs than of NVSL-infected pigs (magnitude indicated by intensity of green color). B) The most significant signaling pathway, “Interferon Signaling”, that included differentially expressed genes for High/Low TVclass. Eleven genes were expressed more in tonsils of High-TVclass pigs than of Low-TVclass pigs (magnitude indicated by intensity of red color). **Figure S7.** The *GBP5* gene was predicted to be more activated or less inhibited, in KS06-infected pigs compared to NVSL-infected pigs based on the tonsil NanoString data when accounting for cell enrichments (*p*=0.009 and z=1.38). Orange/blue color represents activation/inhibition of the functions. The genes in red/green were expressed more/less in tonsil of pigs with the AB than the AA WUR genotype (magnitude indicated by intensity of red or green color). The dashed lines represent indirect interactions; the blunt arrow indicates negative regulation and “➔“ indicates positive regulation.

**Additional file 2: Table S1**. Cell types included in the significant group. **Table S2.** Top three cell types explained by each principal component of cell type enrichments in the significant and the nuisance groups of cell types. **Table S3.** Numbers of differentially expressed genes in tonsil based on the RNA-seq data (q<0.1) with or without accounting for cell enrichments.

## Data Availability

All data generated and/or analyzed in this study are stored in the PHGC relational database http://www.animalgenome.org/lunney/index.php. The data are not publicly available because the they were generated on samples from commercially owned animal but access can be provided upon reasonable request to the authors.

## Abbreviations

AA_KS06: AA genotype pigs infected with KS06
AA_NVSL: AA genotype pigs infected with NVSL
AA_High: AA genotype pigs infected with high virus level in tonsil
AA_Low: AA genotype pigs infected with low virus level in tonsil
AB_KS06: AB genotype pigs infected with KS06
AB_NVSL: AB genotype pigs infected with NVSL
AB_High: AB genotype pigs infected with high virus level in tonsil
AB_Low: AB genotype pigs infected with low virus level in tonsil
BIC: Bayesian information criterion
CE: Cell-type enrichments
+CE: With cell-type enrichments
-CE: Without cell-type enrichments
DE: Differentially expression
DEGs: Differentially expressed genes
dpi: Days post infection
dsRNA: Double-stranded RNA
log_2_FC: log_2_(Fold Change)
FDR: False discovery rate
GBP1: Guanylate binding protein 1 gene
GBP5: Guanylate binding protein 5 gene
HB: Hemoglobin
High-TVclass: High virus levels in tonsil
Low-TVclass: Low virus levels in tonsil
IPA: Ingenuity Pathway Analysis
Isolate +CE-DEGs: Differentially expressed genes for Isolate from the model with cell-type enrichments
Isolate -CE-DEGs: Differentially expressed genes for Isolate from the model without cell-type enrichments
Isolate*WUR + CE-DEGs: Differentially expressed genes for the interaction between Isolate and WUR in the model with cell-type enrichments
Iso-Seq: Single-molecule long-read isoform sequencing
KS06: KS-2006-72109
KS06_High: Pigs infected with KS06 ending with high virus level in tonsil
KS06_Low: Pigs infected with KS06 ending with low virus level in tonsil
MLV: Modified live vaccine
Nui: Nuisance cell type group
NuiPC: Principal components for the nuisance cell type group
NVSL: NVSL-97-7895
NVSL_High: Pigs infected with NVSL PRRSV ending with high virus level in tonsil
NVSL_Low: Pigs infected with NVSL PRRSV ending with low virus level in tonsil
PCA: Principal component analyses
PHGC: PRRS Host Genetics Consortium
PRRS: Porcine reproductive and respiratory syndrome
PRRSV: Porcine reproductive and respiratory syndrome virus
RIN: RNA integrity number
Sig: Significant cell type group
SigPC: Principal components for the significant cell type group
TVclass: Tonsil viral level
VL: Viral load
WG: Weight gain
WUR: SNP rs80800372
