# Supplementary material for: Gene expression in tonsils in swine following infection with porcine reproductive and respiratory syndrome virus
Source: BMC Vet Res. 2021 Feb 22;17:88. doi: 10.1186/s12917-021-02785-1 (PMC7901068; doi:10.1186/s12917-021-02785-1)
Supplement: Supplementary file 1 — Additional file 1: Figure S1. The relationship between RIN score and tonsil viral level in pigs infected with NVSL (A) and KS06 (B). The samples associated with selected samples for RNA-seq (High-TVclass in red; Low-TVclass in green) and unselected samples (in blue) were converted to a color representation. Figure S2. Plots of each two of top three principle components (PC) for the 28 cell types that were significantly(p<0.1) affected by at least one of Isolate, TVclass, WUR genotype, their interactions, sex, or age. The cell compositions in tonsils were predicted based on the tonsil RNA-seq data using the xCell software. Shapes indicate samples from NVSL or KS06-infected pigs. Colors indicate samples with high or low tonsil viral level. PC’s 1, 2, and 3 explained 31.0, 22.0, and 18.4%, respectively, of the variance in cell composition of the 28 cell types. Figure S3. Hierarchical clustering of the 200 most variant genes based on upper quartile normalized read counts. Fifty-one RNA-seq samples were clustered to identify any obvious outliers and potential clustering of samples by factors of interest, using euclidean method to calculate the sample distance matrix, and the average agglomeration method to apply hierarchical clustering. The samples associated with the factors of Isolate (white=NVSL; red=KS06), TVclass (white=High; red=Low), WUR genotype (white=AA; red=AB), sex (white=Female, red=Male), and RIN (low ➔ high = white ➔ red) were converted to a color representation. Figure S4. Significant canonical pathways related to immune response that include differentially expressed genes for isolate in tonsils at 42 days post infection when not accounting for cell enrichments based on the RNA-seq data. The expression values of genes with a false discovery rate <0.1 were used to calculate the z-scores and -log(p-values) for each pathway. Pathways shown in this figure have a -log(p-value)>1.3 and an absolute z-score > 2. The height of each bar corresponds to the -log(p-va [file 12917_2021_2785_MOESM1_ESM.pptx]

## Slide 1
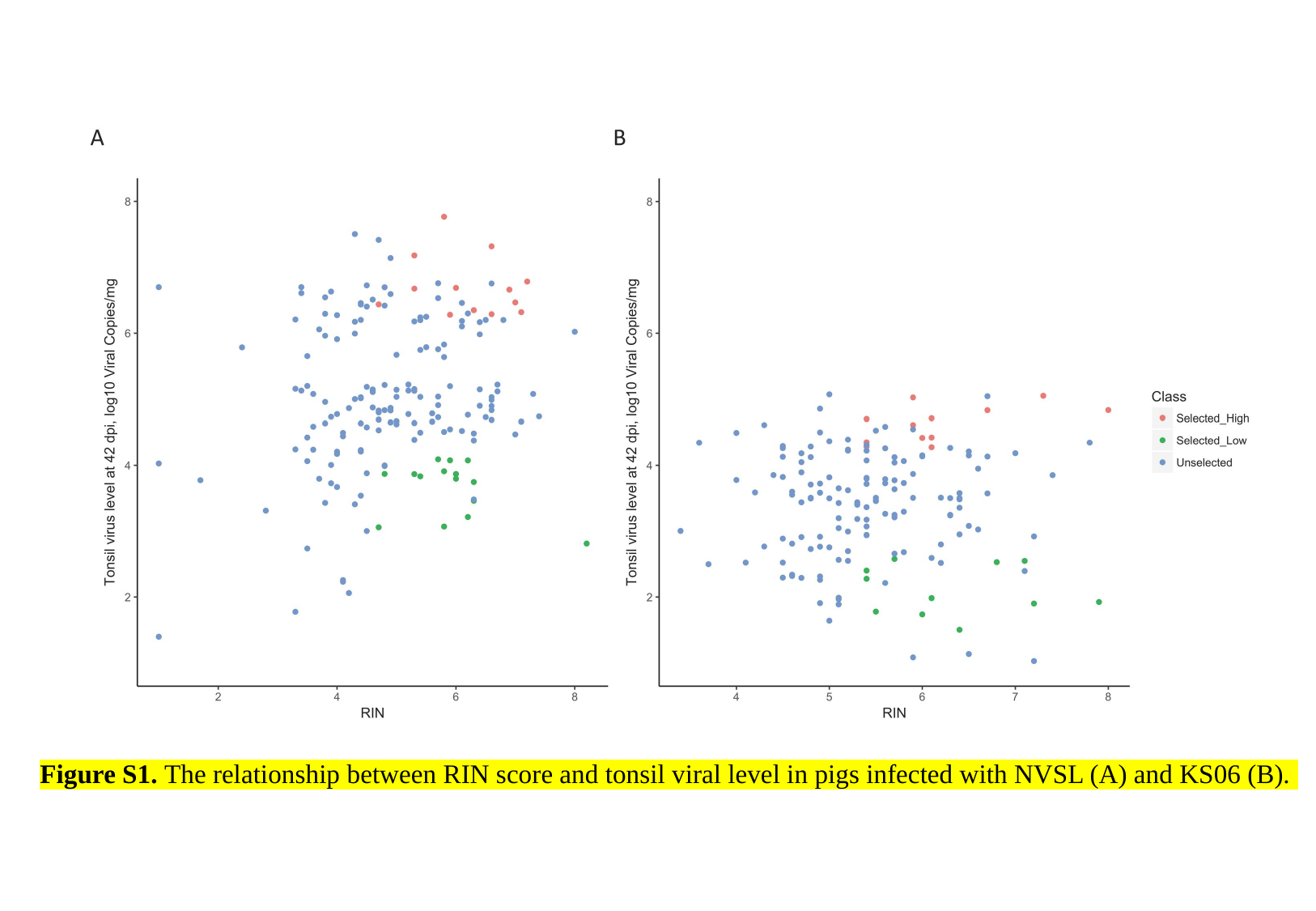

Figure S1. The relationship between RIN score and tonsil viral level in pigs infected with NVSL (A) and KS06 (B).

## Slide 2
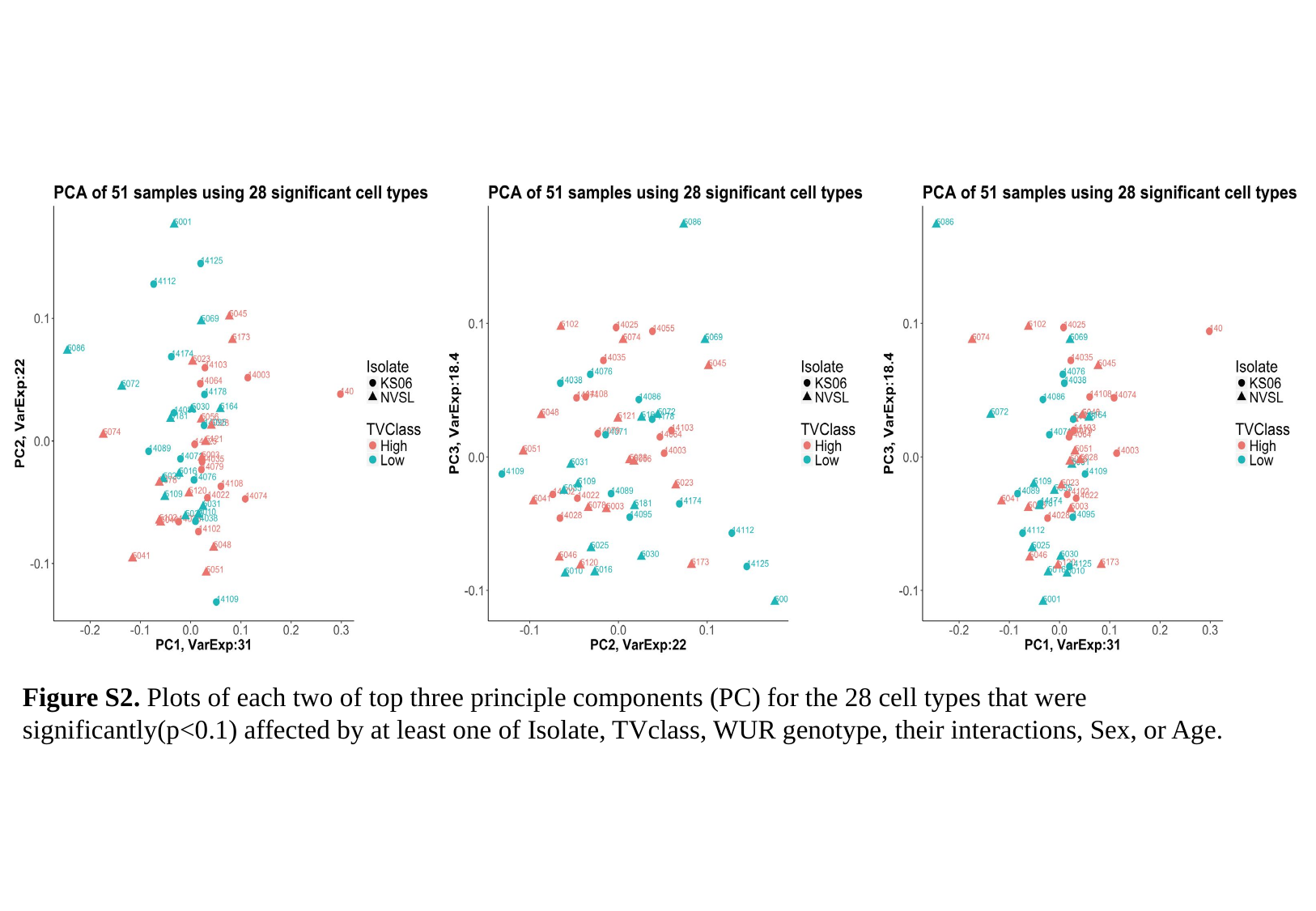

Figure S2. Plots of each two of top three principle components (PC) for the 28 cell types that were significantly(p<0.1) affected by at least one of Isolate, TVclass, WUR genotype, their interactions, Sex, or Age.

## Slide 3
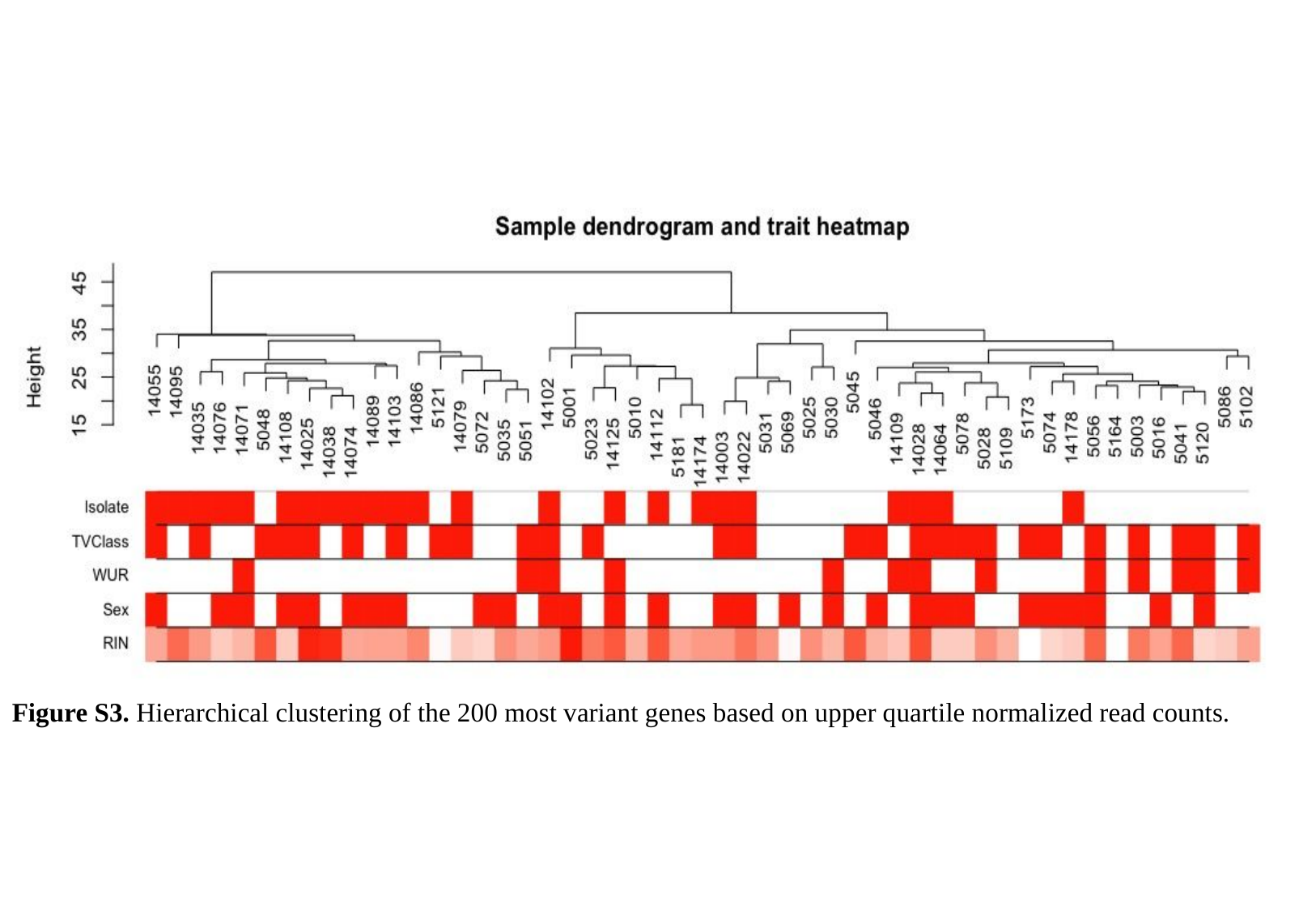

Figure S3. Hierarchical clustering of the 200 most variant genes based on upper quartile normalized read counts.

## Slide 4
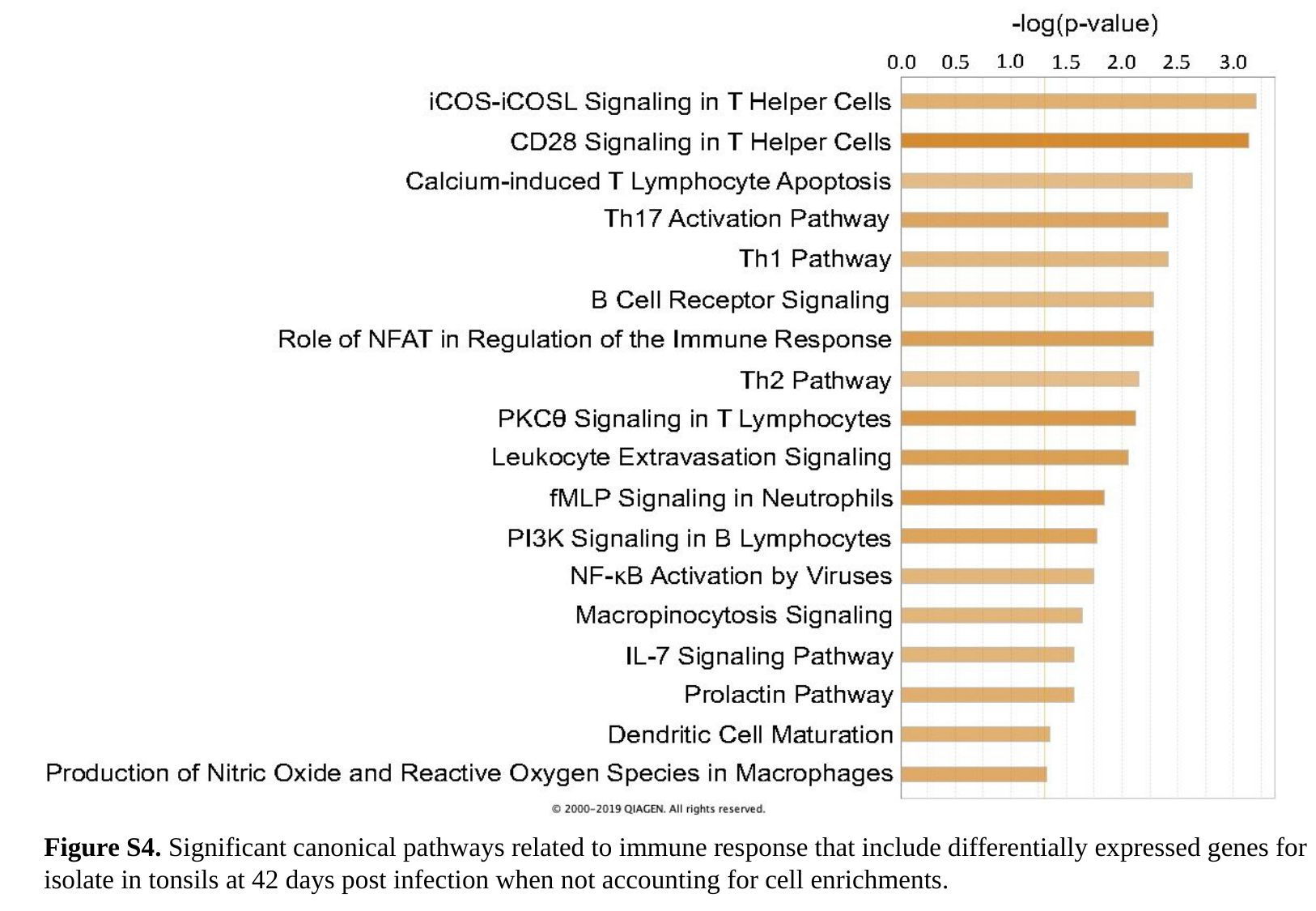

Figure S4. Significant canonical pathways related to immune response that include differentially expressed genes for isolate in tonsils at 42 days post infection when not accounting for cell enrichments.

## Slide 5
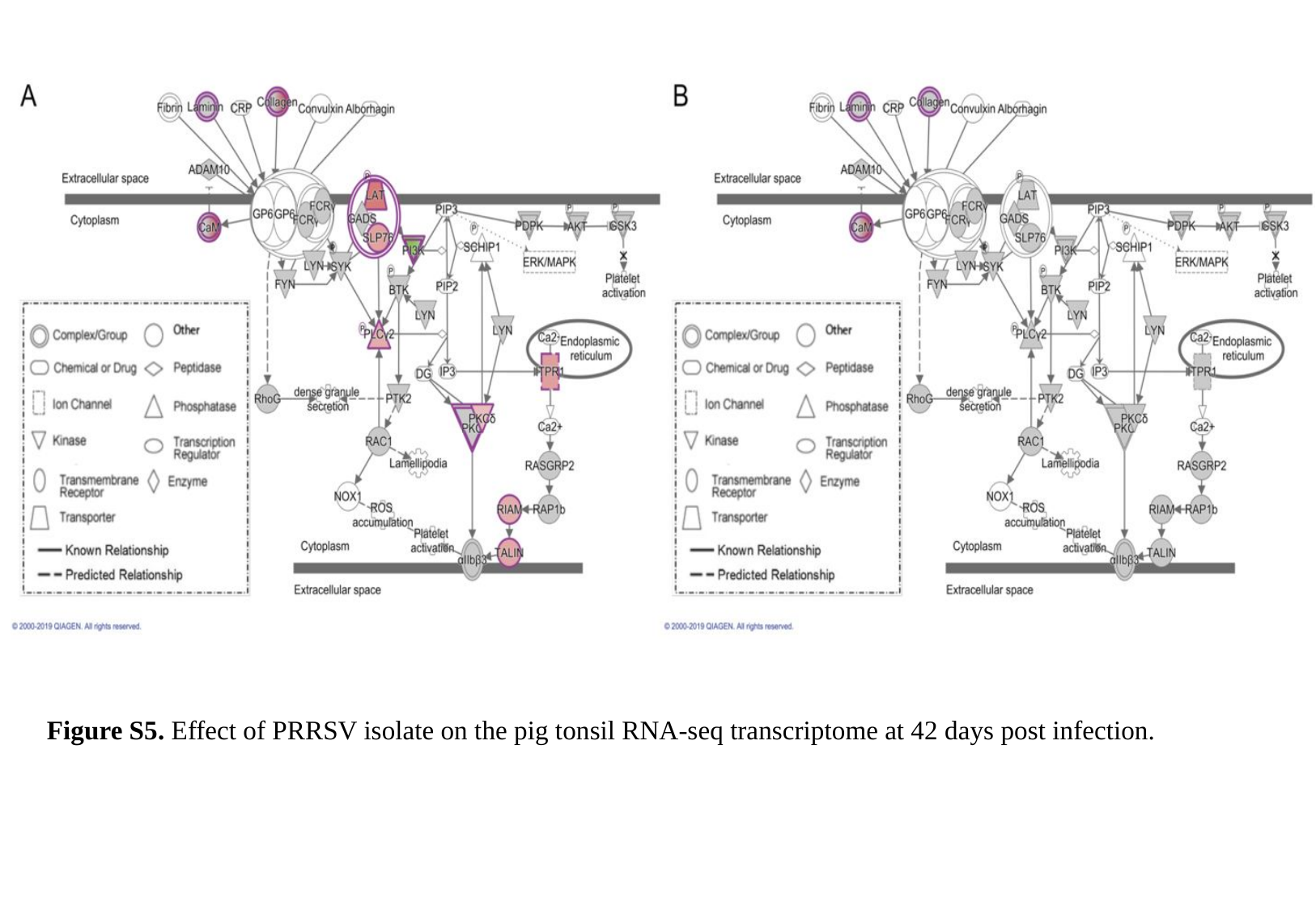

Figure S5. Effect of PRRSV isolate on the pig tonsil RNA-seq transcriptome at 42 days post infection.

## Slide 6
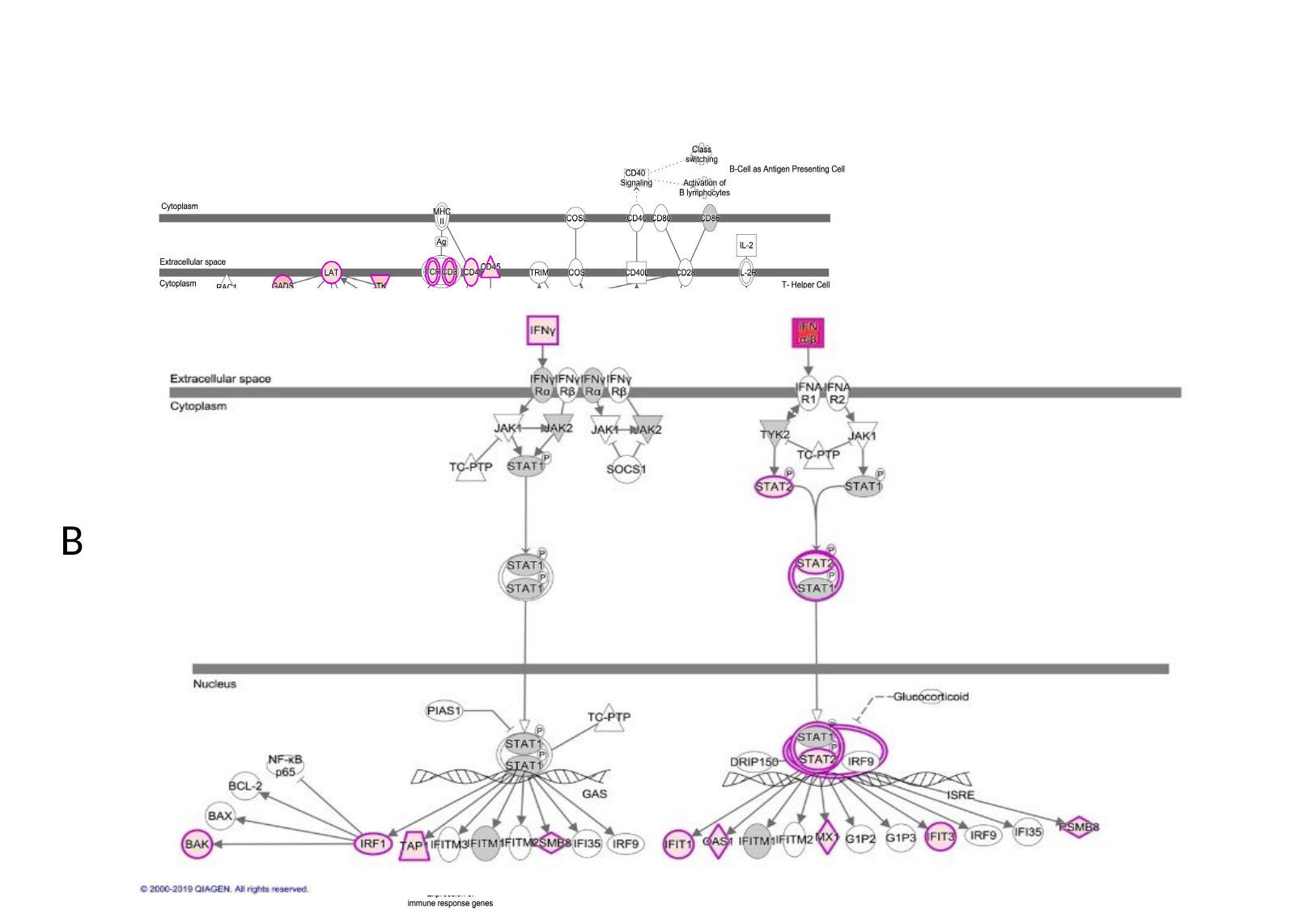

A
B
Figure S6. Effect of PRRSV isolate and TVclass on the expression of 230 immune related genes in tonsil at 42 days post infection based on the NanoString data without accounting for cell enrichments.

## Slide 7
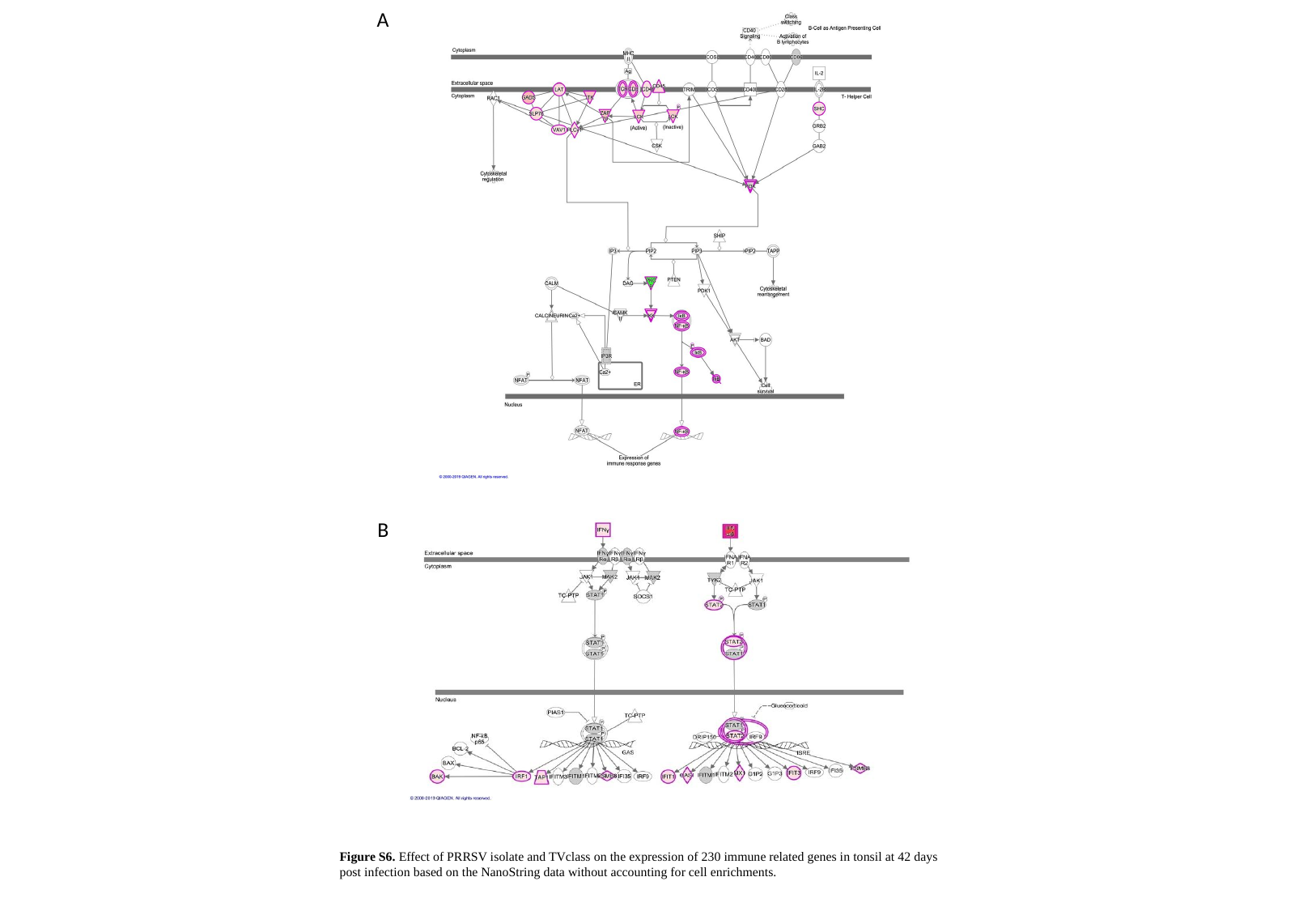

A
B
Figure S6. Effect of PRRSV isolate and TVclass on the expression of 230 immune related genes in tonsil at 42 days post infection based on the NanoString data without accounting for cell enrichments.

## Slide 8
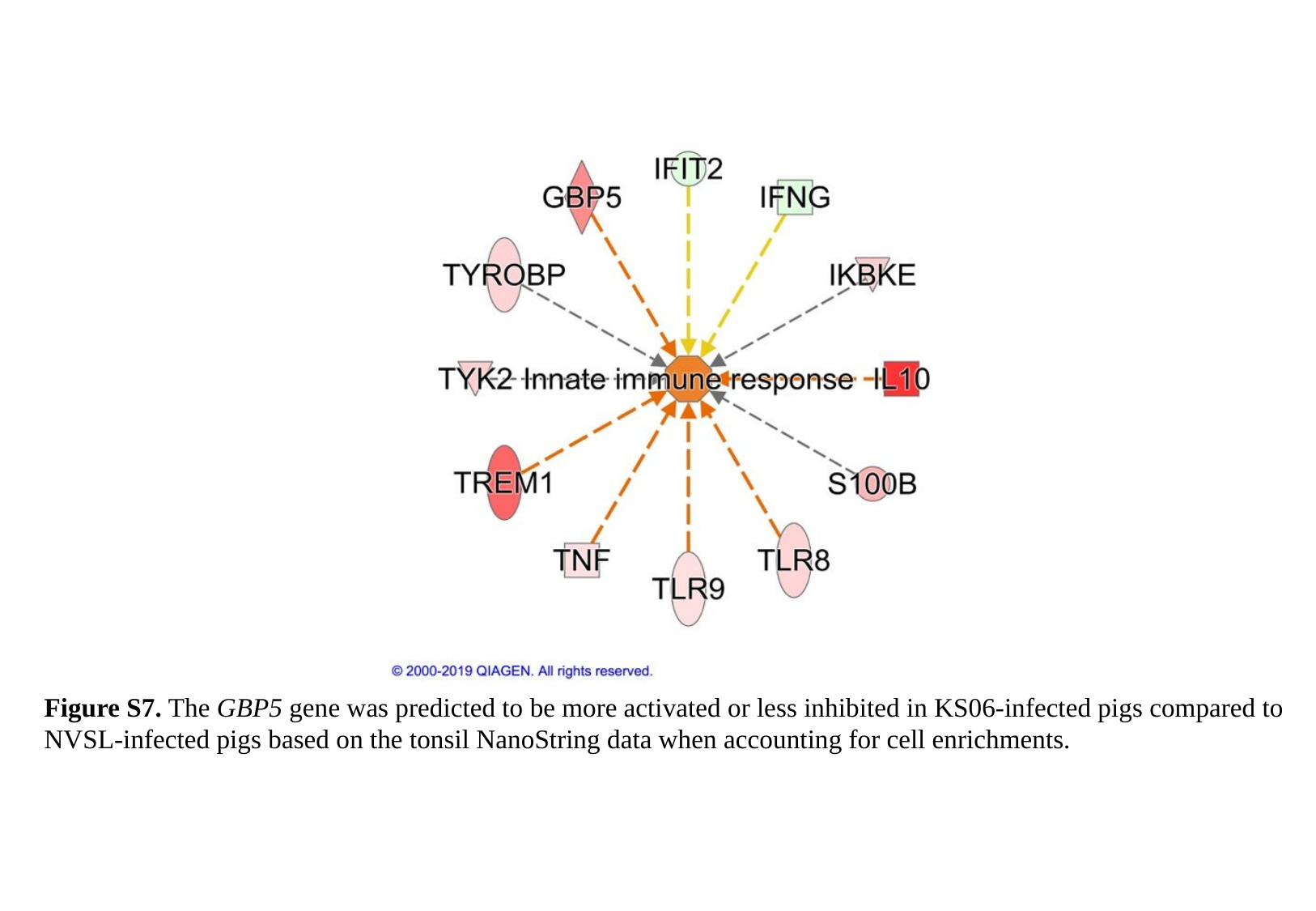

Figure S7. The GBP5 gene was predicted to be more activated or less inhibited in KS06-infected pigs compared to NVSL-infected pigs based on the tonsil NanoString data when accounting for cell enrichments.
