# Supplementary material for: Gene expression in tonsils in swine following infection with porcine reproductive and respiratory syndrome virus
Source: BMC Vet Res. 2021 Feb 22;17:88. doi: 10.1186/s12917-021-02785-1 (PMC7901068; doi:10.1186/s12917-021-02785-1)
Supplement: Supplementary file 2 — Additional file 2: Table S1. Cell types included in the significant group. Table S2. Top three cell types explained by each principal component of cell type enrichments in the significant and the nuisance groups of cell types. Table S3. Numbers of differentially expressed genes in tonsil based on the RNA-seq data (q<0.1) with or without accounting for cell enrichments. [file 12917_2021_2785_MOESM2_ESM.docx]

**Table S1**. Cell types included in the significant group.

| Cell type | Subgroup | Parent/Child | Final_model |
| --- | --- | --- | --- |
| Neurons | Epithelial | Astrocytes | Age |
| Common lymphoid progenitors | HSC | HSC | Isolate |
| Multipotent progenitors | HSC | Parent | Isolate |
| Epithelial cells | Epithelial | Parent | Isolate+RIN+Sex |
| Mesangial cells | Stroma | Fibroblasts | Isolate+TVclass+Isolate*TVclass |
| Plasmacytoid dendritic cells | Myeloid | DC | Isolate+TVclass+WUR |
| Macrophages | Myeloid | Parent | Isolate+TVclass+WUR+ Isolate*TVclass |
| Endothelial cells | Stroma | Parent | Isolate+TVclass+WUR+RIN+Sex+ Isolate*TVclass |
| Microvascular endothelial cells | Stroma | Endothelial cells | Isolate+TVclass+WUR+Sex+ Isolate*TVclass |
| CD8+ T-cells | Lymphoid | Parent | Isolate+WUR+Isolate*WUR |
| Myocytes | Stroma | Skeletal muscle | Isolate+WUR+RIN+Isolate*WUR |
| B-cells | Lymphoid | Parent | Sex |
| Macrophages M2 | Myeloid | Macrophages | Sex |
| Neutrophils | Myeloid | Parent | Sex |
| naive B-cells | Lymphoid | B-cells | Sex+Age |
| CD8+ naive T-cells | Lymphoid | CD8+ T-cells | TVclass |
| Class-switched memory B-cells | Lymphoid | B-cells | TVclass |
| Keratinocytes | Epithelial | Epithelial cells | TVclass |
| Plasma cells | Lymphoid | B-cells | TVclass |
| Basophils | Myeloid | Parent | TVclass+Age |
| CD8+ central memory T-cells | Lymphoid | CD8+ T-cells | TVclass+Sex |
| Type 1 T-helper cells | Lymphoid | CD4+ T-cells | TVclass+WUR |
| Eosinophils | Myeloid | Parent | TVclass+WUR+TVclass*WUR |
| Lymphatic endothelial cells | Stroma | Endothelial cells | TVclass+WUR+TVclass*WUR |
| Mesenchymal stem cells | Stroma | Parent | TVclass+WUR+TVclass*WUR |
| Fibroblasts | Stroma | Parent | WUR |
| Hematopoietic stem cells | HSC | Parent | WUR |
| Megakaryocyte_erythroid progenitors | HSC | HSC | WUR+Sex |

**Table S2**. Top three cell types explained by each principal component of cell type enrichments in the significant and the nuisance groups of cell types.

| Principle component | Top three cell types |
| --- | --- |
| SigPC1^a^ | Epithelial cells, B cells, and microvascular endothelial cells |
| SigPC2 | Keratinocytes, mesangial cells, and myocytes |
| SigPC3 | Macrophages M2, neurons, and megakaryocytes |
| NuiPC1^b^ | CD8+ central memory T-cells, CD4+ memory T-cells, CD4+ T-cells |
| NuiPC2 | Immature dendritic cells, chondrocytes, platelets |
| NuiPC3 | Common myeloid progenitors, mast cells, sebocytes |

^a^ The first principle component of the significant cell group;

^b^ The first principle component of the nuisance cell group.

**Table S3**. Numbers of differentially expressed genes in tonsil based on the RNA-seq data (q<0.1) with or without accounting for cell enrichments.

| Factor | Accounting for cell enrichments | | Overlap |
| --- | --- | --- | --- |
|  | No | Yes |  |
| Isolate: KS06/NVSL | 1074 | 204 | 162 |
| TVclass: Low/High | 0 | 4 | 0 |
| WUR: AB/AA | 0 | 0 | 0 |
| Isolate*WUR | - | 107 | - |
| TVclass*WUR | - | 5 | - |
| Sex | 25 | 26 | 24 |
| RIN | 3824 | 461 | 437 |
| SigPC1^a^ | - | 1637 | - |
| SigPC2 | - | 10275 | - |
| SigPC3 | - | 238 | - |
| NuiPC1^b^ | - | 4193 | - |

^a^ The first principle component of the significant cell group;

^b^ The first principle component of the nuisance cell group.
